# Omics Analyses of Trichoderma reesei CBS999.97 and QM6a Indicate the Relevance of Female Fertility to Carbohydrate-Active Enzyme and Transporter Levels

**DOI:** 10.1128/AEM.01578-17

**Published:** 2017-10-31

**Authors:** Doris Tisch, Kyle R. Pomraning, James R. Collett, Michael Freitag, Scott E. Baker, Chia-Ling Chen, Paul Wei-Che Hsu, Yu Chien Chuang, Andre Schuster, Christoph Dattenböck, Eva Stappler, Michael Sulyok, Stefan Böhmdorfer, Josua Oberlerchner, Ting-Fang Wang, Monika Schmoll

**Affiliations:** a TU Wien, Insitute of Chemical Engineering, Research Area Molecular Biotechnology, Vienna, Austria; b Pacific Northwest National Laboratory, Richland, Washington, USA; c Oregon State University, Department of Biochemistry and Biophysics, Corvallis, Oregon, USA; d Academia Sinica, Institute of Molecular Biology, Taipei, Taiwan; e AIT Austrian Institute of Technology, Department Health and Environment—Bioresources, Tulln, Austria; f University of Natural Resources and Life Sciences BOKU, Department of Agrobiotechnology, Center for Analytical Chemistry, Tulln, Austria; g University of Natural Resources and Life Sciences Vienna, Department of Chemistry, Division of Chemistry of Renewable Resources, Tulln, Austria; University of Bayreuth

**Keywords:** Trichoderma reesei, Hypocrea jecorina, cellulase, strain improvement, sexual development, female fertility, secondary metabolism, mating type, carbon source

## Abstract

The filamentous fungus Trichoderma reesei is found predominantly in the tropics but also in more temperate regions, such as Europe, and is widely known as a producer of large amounts of plant cell wall-degrading enzymes. We sequenced the genome of the sexually competent isolate CBS999.97, which is phenotypically different from the female sterile strain QM6a but can cross sexually with QM6a. Transcriptome data for growth on cellulose showed that entire carbohydrate-active enzyme (CAZyme) families are consistently differentially regulated between these strains. We evaluated backcrossed strains of both mating types, which acquired female fertility from CBS999.97 but maintained a mostly QM6a genetic background, and we could thereby distinguish between the effects of strain background and female fertility or mating type. We found clear regulatory differences associated with female fertility and female sterility, including regulation of CAZyme and transporter genes. Analysis of carbon source utilization, transcriptomes, and secondary metabolites in these strains revealed that only a few changes in gene regulation are consistently correlated with different mating types. Different strain backgrounds (QM6a versus CBS999.97) resulted in the most significant alterations in the transcriptomes and in carbon source utilization, with decreased growth of CBS999.97 on several amino acids (for example proline or alanine), which further correlated with the downregulation of genes involved in the respective pathways. In combination, our findings support a role of fertility-associated processes in physiology and gene regulation and are of high relevance for the use of sexual crossing in combining the characteristics of two compatible strains or quantitative trait locus (QTL) analysis.

**IMPORTANCE** Trichoderma reesei is a filamentous fungus with a high potential for secretion of plant cell wall-degrading enzymes. We sequenced the genome of the fully fertile field isolate CBS999.97 and analyzed its gene regulation characteristics in comparison with the commonly used laboratory wild-type strain QM6a, which is not female fertile. Additionally, we also evaluated fully fertile strains with genotypes very close to that of QM6a in order to distinguish between strain-specific and fertility-specific characteristics. We found that QM6a and CBS999.97 clearly differ in their growth patterns on different carbon sources, CAZyme gene regulation, and secondary metabolism. Importantly, we found altered regulation of 90 genes associated with female fertility, including CAZyme genes and transporter genes, but only minor mating type-dependent differences. Hence, when using sexual crossing in research and for strain improvement, it is important to consider female fertile and female sterile strains for comparison with QM6a and to achieve optimal performance.

## INTRODUCTION

Competition in nature drives adaptation of physiological processes to the specific conditions in the microbial habitats. Thus, abilities to economically respond to the availability and nature of substrates to sustain growth, as well as to changing light conditions, are among the most crucial competencies for survival and evolutionary success.

Trichoderma reesei is a prolific producer of cellulases and is broadly utilized in research and industry (1, 2). Its physiological abilities and the tools available for work with T. reesei, such as efficient transformation, including several markers, its genome sequence, and a high-throughput gene deletion strategy, make it a model for carbon metabolism and cellulase gene regulation (3–5), as well as for light response and signaling (6). The connections between these pathways have provided new approaches for industrial strain improvement (7).

The genome of T. reesei encodes a relatively small set of cellulases and hemicellulases compared to the genomes of other fungi (8). In many cases, carbohydrate-active enzymes (CAZymes) are arranged in clusters in the genome, and some of these clusters contain polyketide synthetases (PKS) or nonribosomal peptide synthetases (NRPS) (8). For industrial application of T. reesei, the ability to produce secondary metabolites is of high importance. Recently, connections between primary and secondary metabolism were indeed shown (9, 10). Comparison of the genome of T. reesei with the genomes of the potent biocontrol fungi Trichoderma atroviride and Trichoderma virens indicated that at least part of the mycoparasitism-specific gene content was lost in evolution in T. reesei (5, 11). However, the genome of T. reesei contains expanded gene families that may contribute to high levels of cellulase gene expression and heterologous protein production (5).

Sexual development of T. reesei under laboratory conditions was only achieved in 2009 (12). The coincidental identification of a peptide pheromone precursor gene in T. reesei (13, 14) had led to the evaluation of mating capabilities and consequently to the discovery of sexual development and the ability to produce sexually fertile progeny. CBS999.97, a strain isolated in French Guiana (15), can produce fruiting bodies on plates and was evaluated in parallel with strain QM6a, which is the parental strain of all industrially used mutants (12). QM6a is female sterile, in contrast to CBS999.97, which strongly increased the importance of CBS999.97 for industrial strain improvement. CBS999.97 was used to reintroduce the capability to mate into the cellulase-enhanced mutant strain QM9414 by backcrossing (16). Later, the same method was used to obtain the female fertile strains FF1 and FF2, which carry different mating type alleles in a QM6a background (17). Recently, CBS999.97 was used to identify the mutation responsible for female sterility of QM6a (18).

In the course of sexual development, T. reesei forms 16 ascospores by meiosis and postmeiotic mitoses (19). Interestingly, sequence heterozygosity between QM6a and its derivatives and CBS999.97 results in a considerable number of inviable ascospores, as well as segmentally aneuploid ascospores: more than 90% of asci contain, at least in part, inviable ascospores. The aneuploidy increases the levels of xylanases and loss of spore pigmentation. These changes are assumed to provide a transient selective advantage for growth on lignocellulosic biomass, which is lost after roughly 2 weeks (19) due to an as-yet-unknown mechanism. The diversity within T. reesei as reflected by strains QM6a and CBS999.97 indicates a rich source for strain modification and quantitative trait locus (QTL) analysis, with sexual development as a challenging but also promising tool.

**FIG 1:**
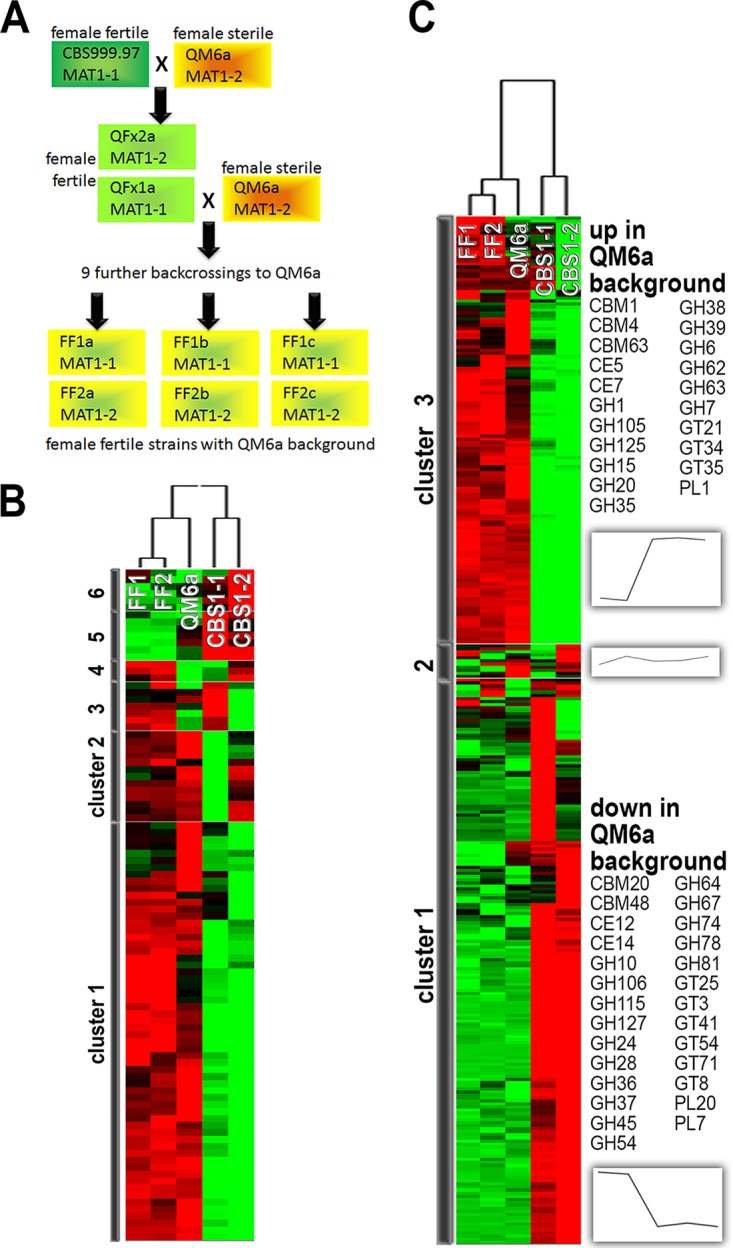
Strains and their metabolic and regulatory characteristics. (A) CBS999.97 and QM6a are original isolates (15, 55). As the CBS999.97 isolate consisted of a mixture of two strains of different mating types, they were separated to obtain compatible strains of both mating types, designated CBS999.97 MAT1-1 and CBS999.97 MAT1-2 (12). Female fertile CBS999.97 MAT1-1 was used for backcrossing with female sterile QM6a in order to obtain female fertile strains showing the physiological characteristics of QM6a (FF1a, FF1b, and FF1c). FF1a, FF1b, and FF1c were constructed in the same backcrossing procedure, with 10 backcrossings in total, but are progeny of individual separate crossing lines of CBS999.97 MAT1-1 (17). (B) Hierarchical cluster analysis of growth on diverse carbon sources. Growth of QM6a (MAT1-2), FF1a, -b, and -c (female fertile sister strains of mating type MAT1-1, treated as replicates), and FF2a, -b, and -c (female fertile sister strains of mating type MAT1-2, treated as replicates) was analyzed on 95 carbon sources after 72 h. Hierarchical clustering was done for both strain background and carbon source using HCE3.5. Carbon sources with similar expression patterns across strain backgrounds are shown close to each other. (C) Regulation patterns of CAZyme-encoding genes in different genetic backgrounds. Transcript levels of CAZyme genes from CBS999.97 of both mating types and from strains QM6a, FF1, and FF2 were analyzed by hierarchical clustering. CAZyme gene families of which all members present in the QM6a genome are upregulated in strains with the QM6a background (QM6a, FF1, and FF2) are shown as cluster 3, and those with all members downregulated in QM6a background strains compared to their expression in CBS999.97 of both mating types are shown as cluster 1 (see also Data Set S3 in the supplemental material).

Considering sexual development as a tool in industry, it is required that this process work reliably and continuously. However, it was shown for other fungi that asexual cultivation, which is routinely done for inoculum generation, results in the loss of female fertility (20, 21). Also, for T. reesei QM6a, it was considered possible that its defect might be a result of repetitive laboratory cultivation (12). We evaluated the wild-type strains QM6a and CBS999.97, as well as female fertile strains after 10 rounds of backcrossing of CBS999.97 with QM6a (Fig. 1A), as a comparable approach would also be valuable to create female fertile production strains. We found considerable differences in transcript regulation patterns and carbon source utilization depending on strain background and female fertility. Our results show that notable changes in metabolic capacities, including CAZyme expression and substrate degradation, have to be expected in progeny from crosses between strains in the QM6a genetic background and those in the CBS999.97 background.

## RESULTS

### Genome comparisons and distribution of SNPs.

The genome sequence of strain CBS999.97 was assembled from 454 and paired-end Illumina reads to generate 1,087 contigs that were ordered into 167 scaffolds, totaling 32.47 Mb, with an N50 of 1.22 Mb. Alignment of the high-throughput-sequencing reads from CBS999.97 to the QM6a reference genome identified 466,083 single nucleotide polymorphisms (SNPs) between the strains, 36,955 of which are predicted to be nonsynonymous (data available at https://doi.org/10.13140/RG.2.2.26804.45449 and in Data Set S1 in the supplemental material).

The SNPs were not randomly distributed among genes or gene groups. The 2,390 predicted genes with no nonsynonymous SNPs are strongly enriched in core physiological functions. The most strongly enriched functions include “metabolism” (*P* = 0.0), “stress response” (*P* = 2.23 × 10^−13^), “energy” (*P* = 1.54 × 10^−11^), “protein synthesis” (*P* = 2.05 × 10^−10^), “regulation of C compound and carbohydrate metabolism” (*P* = 1.25 × 10^−7^), “fungal-cell-type differentiation” (*P* = 2.11 × 10^−6^), “signal transduction mechanisms” (*P* = 2.11 × 10^−6^), and “transcription” (*P* = 8.62 × 10^−5^) (Data Set S1). In contrast, genes containing more than 10 nonsynonymous SNPs are enriched in “secondary metabolism” (*P* = 4.91 × 10^−7^), “DNA repair” (*P* = 1.26 × 10^−3^), and “photoperception and -response” (*P* = 4.45 × 10^−3^) (Data Set S1).

We also checked for genomic regions with increased occurrence of nonsynonymous SNPs, which might indicate chromosomal areas under specific evolutionary pressure. Regions with 5 or more genes in close vicinity in the genome sequence containing 10 nonsynonymous SNPs were considered. Indeed, we found 19 regions that fit this definition of being under evolutionary pressure (Data Set S1). In several cases, these regions are located at the ends of scaffolds, indicating that they are close to repetitive regions. Checking overlaps with CAZyme clusters, we only found the region around TR_107835, an as-yet-uncharacterized carbohydrate esterase of family 10, to reside within a region of increased abundance of nonsynonymous SNPs. This result is consistent with the finding that metabolic functions are enriched among genes with a particularly low abundance of nonsynonymous SNPs. Interestingly, *ck1d*/TR_55049, which encodes a casein kinase I (CKI), representing an additional CKI present in T. reesei but not in T. atroviride, T. virens, or Neurospora crassa (5), is also found in a region with few nonsynonymous SNPs, which may indicate evolutionary conservation for this gene or functions of casein kinases I in general in T. reesei. Clustering of CAZyme genes in QM6a (8) was largely retained in CBS999.97, except for the chitinase gene *chi18-16*, which was relocated in CBS999.97, and one split cluster (for details see Note S1, Fig. S1, and Data Set S2).

### Restoration of female fertility by backcrossing of QM6a.

In order to be able to evaluate the consequences of backcrossing for reintroduction of female fertility, we constructed strains by 10 rounds of backcrossing with QM6a and selection for mating type and female fertility (Fig. 1A). We obtained three progeny of independent breeding lines with MAT1-1 that we named FF1a, FF1b, and FF1c. They were sequenced and mapped onto the QM6a genome in order to identify regions retained from CBS999.97 by increased SNP abundance (Table S1). Three genomic regions present in all three independent progeny were found in scaffolds 2, 6, and 21 of the published QM6a genome (http://genome.jgi.doe.gov/Trire2/Trire2.home.html) (8).

Since the distribution of female fertile and female sterile progeny from our crosses was roughly 50:50, we assumed that a mutation in a single gene or at least alteration in a single genomic locus should be responsible. According to the equations put forward by Leslie (22) for inbreeding without a selected marker, the expected proportion of the genome retained from CBS999.97 in the case of a single region being responsible for the selected trait would be 0.5^10^. Considering the 9,127 genes predicted for the genome, this would mean 9 genes would be expected to differ between FF1a, -b, and -c and QM6a. However, the sequence analysis revealed 106 genes from CBS999.97 after 10 backcrossing steps—a considerably higher number of consistently retained genes in overlapping regions (Table S1). Therefore, we performed an additional backcrossing round and selected 20 female fertile and 20 female sterile strains. We chose 17 SNP-containing genes representing all three overlapping genomic regions and analyzed whether the CBS999.97 or the QM6a version of the respective gene was present in these progeny. This analysis clearly showed a significant correlation of female fertility with the presence of the CBS999.97 version of six genes encoding factors designated FS4, FS5, FS6, FS7, FS8, and FS9 on scaffold 6 and no significant correlation with the genes on the other scaffolds (Table S2). This finding was in line with our hypothesis of one locus being responsible for the female fertility defect in QM6a. Consequently, the genomic region spanning the loci of these genes was found to be responsible for female fertility in the backcrossing strains and to contain the defective gene in QM6a (23). Later on, FS5 (TR_67350) was shown to harbor the mutation that caused female sterility of QM6a (18).

Previously, segmental aneuploidy was reported for T. reesei (19), which could influence the efficacy of the inbreeding process (24–26). However, our genome sequence analysis showed that the loci where aneuploidy occurs (19) and the region responsible for female sterility in QM6a are not related.

### Metabolic capacities in QM6a and CBS999.97 backgrounds.

CBS999.97 and QM6a differ considerably upon growth on solid medium (12). We evaluated the metabolic capacities of strains with CBS999.97 and QM6a backgrounds, as well as any relevance of the ability to undergo sexual development in the regulation of metabolic processes, with the Biolog system. We investigated the growth, as reflected by turbidity measurements, of both mating types of CBS999.97, as well as FF1, FF2, and QM6a, on 96 carbon sources in multiwell plates. For FF1 and FF2, we additionally used two sister strains each from different individual backcrossing lines, resulting in three independent female fertile strains with the QM6a background for both mating types (FF1a, FF1b, and FF1c and FF2a, FF2b, and FF2c) (Fig. 1A).

Hierarchical cluster analysis was used to analyze similarities of the different strains in terms of growth on diverse carbon sources. Strains within a cluster showed similar utilization capabilities for the carbon sources tested. With respect to carbon sources, those that showed consistent patterns across strains were assigned to clusters. The growth patterns of CBS999.97 MAT1-1, CBS999.97 MAT1-2, FF1, FF2, and QM6a (MAT1-2) showed considerable differences between strains with the CBS999.97 background and all strains with the QM6a background (Fig. 1B). This analysis confirmed that after 10 rounds of backcrossing, FF1 (MAT1-1) and FF2 (MAT1-2) strains had largely regained the QM6a phenotype while having acquired female fertility from the first cross with CBS999.97. Variations in carbon source utilization in the FF1 and FF2 strains were minor (Fig. 1B).

### Mating type and carbon utilization in T. reesei.

Mating type can regulate genes associated with metabolism, including sugar transporters (27), in Podospora anserina. Therefore, we were interested in whether any mating type-specific differences in substrate utilization would be consistent in the two phenotypically different strain backgrounds of T. reesei QM6a and CBS999.97. The general strain comparison shown in Fig. 1B already indicates that the different strain backgrounds caused more severe alterations in substrate utilization than did differences in mating types (Fig. 1B) within the individual background. However, some differences were observed between the FF1 and FF2 strains, as well as between CBS999.97 MAT1-1 and CBS999.97 MAT1-2. Detailed comparison of FF1 (MAT1-1) and CBS999.97 MAT1-1 with QM6a, FF2, and CBS999.97 MAT1-2 (all MAT1-2) should reveal if there was any carbon source on which consistent differences between MAT1-1 and MAT1-2 were present. The high standard deviations observed in this analysis are consistent with the strain background being more important than the mating type, as individual replicates matched very well. For MAT1-1 strains, growth was consistently slightly increased relative to the growth of MAT1-2 strains on *N*-acetyl-l-glutamic acid, β-hydroxybutyric acid, and 2-aminoethanol (Fig. S2). Slightly better growth was observed for MAT1-2 strains on α-d-lactose, α-d-glucose, d-arabitol, d-mannose, l-pyroglutamic acid, l-glutamic acid, and interestingly, also on d-cellobiose and d-trehalose (Fig. S2). As several of the latter substrates are associated with cellulose or hemicellulose degradation, relevance of mating type for efficient plant cell wall degradation cannot be excluded.

### The strain background is important for carbon utilization characteristics.

We wanted to evaluate the different strain backgrounds in more detail and so compared CBS999.97 MAT1-1 and CBS999.97 MAT1-2 with QM6a, FF1, and FF2 (Fig. 1B; Fig. S3) (17). CBS999.97 grew better than QM6a on several carbon sources, especially quinic acid (41% ± 7% [mean ± standard deviation] for growth of QM6a compared to that of CBS999.97), which influences several metabolic processes, including (aromatic) amino acid metabolism and transport and sugar transport (28). In contrast, strains with the QM6a background grew better on several amino acids, including l-proline (158% ± 25%); l-proline can be used as a carbon or a nitrogen source and several intermediates of proline metabolism are either shared or feed directly into arginine and glutamate metabolism (29). l-Aspartic acid (137% ± 11%), l-alanine (144% ± 8%), l-serine (148% ± 8%), and l-asparagine (166% ± 11%) also increased the growth of QM6a relative to that of CBS999.97. d-Galacturonic acid (270% ± 88%) was especially efficiently metabolized, which is consistent with the enhanced capability to degrade hemicellulose and/or pectin. Better growth on carbon sources that are building blocks of hemicellulose and metabolites of its degradation (e.g., xylitol [156% ± 18%], d-arabinose [133% ± 18%], and d-galactose [125% ± 10%]) was also observed for QM6a (see also Fig. S3).

### Relationship between female fertility and carbon utilization.

We compared QM6a with the sexually fully competent strains of both mating types (FF1 and FF2) in order to evaluate if there was any metabolic competence that was associated with the genes retained from CBS999.97 during backcrossing. Some of the genes reside in a genomic cluster correlating with female fertility of FF1 and FF2, as discussed above.

The female fertile FF1 and FF2 strains grew better than the female sterile QM6a on d-galacturonic acid (190% ± 17%), xylitol (117% ± 3%), l-rhamnose (125% ± 6%), d-arabinose (147% ± 4%), and l-arabinose (119% ± 2%) (Fig. S4). The latter carbon sources are constituents of or metabolites in the degradative pathway of hemicellulose via the pentose phosphate pathway, suggesting differences in this pathway between QM6a and the backcrossed strains. With respect to amino acids, only growth on l-proline (139% ± 3%) clearly increased in QM6a, while growth on the other amino acids was not significantly different. As hemicellulose metabolism is enhanced in the female fertile FF strains and in strains with the QM6a background relative to that of CBS999.97, we conclude that the differences in hemicellulose metabolism are due to differences in background rather than to differences to female fertility.

### Transcriptome analysis of CBS999.97 and QM6a.

The availability of the female fertile FF1 and FF2 strains with a largely restored background of QM6a enabled evaluation of the impact of full sexual competence on gene regulation upon growth on cellulose. We chose constant darkness as a condition for our experiment, as this most closely resembles the conditions in an industrial fermentor. Additionally, we were interested in how conserved the gene regulation would be in different strains of T. reesei, and hence, we included in our analysis the two strains derived from the wild isolate, CBS999.97 MAT1-1 and CBS999.97 MAT1-2 (12), as well as FF1 and FF2 strains.

### CAZyme gene regulation patterns.

We evaluated the CAZyme gene expression of all annotated CAZymes (5) in CBS999.97 in comparison to their expression in strains with the QM6a background by hierarchical clustering of patterns. In agreement with the substrate utilization patterns observed in the Biolog experiment described above, CAZyme regulation patterns were correlated with the strain background (Fig. 1C). Both increased and decreased transcript levels were observed in QM6a-derived strains relative to the respective transcript levels in the CBS999.97-derived strains. The regulation patterns observed identified three hierarchical clusters: cluster 1 contains 183 genes upregulated in the CBS999.97 background, cluster 2 contains 11 genes showing only minor expression differences between the two strain backgrounds, and cluster 3 contains 139 genes upregulated in the QM6a background (Data Set S3).

The genes for some specific CAZyme families are found only in one of the clusters, suggesting enhanced relevance of particular CAZyme families to the physiology of CBS999.97 or QM6a. Of the carbohydrate binding module (CBM) families, CBM13 was found in all three clusters, while all genes representing CBM20 (binding to cyclodextrins, also known as starch binding domains) and CBM48 (binding to glycogen) were in cluster 1, i.e., their expression was increased in CBS999.97. As both bound substrates may be involved in storage and energy metabolism, these functions are likely to be important for CBS999.97's ability to grow on cellulose. Accordingly, functional category (FunCat) analysis also revealed an enrichment of genes involved in “metabolism of energy reserves” (*P* = 1.8 × 10^−11^) among the genes of cluster 1. In contrast, the expression of genes representing CBM1, CBM4, and CBM63 increased in QM6a. CAZymes in these families were found to bind xylan, different glucans, and amorphous cellulose.

The expression of both putative pectin acetylesterases of carbohydrate esterase (CE) family 12 is upregulated in CBS999.97. In contrast, the expression of the seven genes in CE family 5 and CE family 7 increased in the QM6a background. These gene families encode acetyl xylan esterases acting on xylan, among other substrates. This finding is in agreement with the enhanced regulation of CBM4 in this background, which is involved in binding xylan, among other substrates. Additionally, better growth was observed on carbon sources representing hemicellulose building blocks in the Biolog assay (Fig. S3).

In the glycoside hydrolase families, up- and downregulation often occurred within the groups of regulated genes in a given strain background, reflecting fine-tuning of a specific degradative function. The expression of all four members of glycoside hydrolase (GH) family 28, which are involved in galacturonan degradation, increased in CBS999.97. In contrast, the results of the Biolog assay suggest that enhanced degradation of pectin occurred in strains in the QM6a background, indicating that regulation of the necessary enzymes in QM6a might be better tailored to the utilization of pectin as a substrate. Accordingly, several transcription factors for which a function in plant cell wall degradation was shown are differentially regulated between QM6a and CBS999.97 (for details, see Note S2, Fig. S5, and Data Set S4).

### Mating type-specific gene regulation.

In the analysis of differential gene expression between MAT1-1 and MAT1-2, we found the expected differential regulation of the pheromone receptor genes *hpr1* (upregulated in MAT1-1) and *hpr2* (upregulated in MAT1-2). Only 3 other genes were differentially transcribed more than 2-fold (*P* < 0.01). The expression of TR_121284, encoding an as-yet-uncharacterized protein related to a Saccharomyces cerevisiae N-terminal amidase that is involved in the N-end rule protein degradation pathway, is considerably reduced in MAT1-1. The expression of TR_121499, encoding a SANT family protein, and TR_106178, encoding a putative RNA binding protein, was also reduced in MAT1-1 strains. As no CAZyme genes or other characterized metabolism-related genes were significantly differentially regulated between MAT1-1 strains and MAT1-2 strains, no major differences are predicted for substrate degradation by strains with similar genetic backgrounds but different mating types.

### Differential gene regulation between strains with the QM6a background and CBS999.97.

We found considerable differences in gene regulation between strains with the QM6a and CBS999.97 backgrounds, and we set a more stringent threshold of 5-fold differential expression with a *P* value of <0.01. Applying this criterion, we found 894 downregulated genes and 424 upregulated genes in strains with the CBS999.97 background relative to those with the QM6a background (Data Set S5).

**FIG 2:**
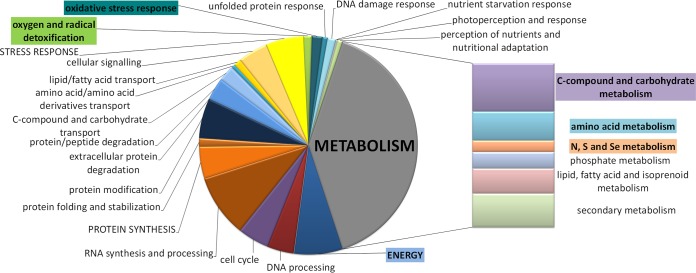
Functional categories of genes upregulated in the QM6a background. Genes upregulated in the QM6a genetic background (strains QM6a, FF1, and FF2) compared to their expression in CBS999.97 of both mating types were subjected to functional category analysis (Data Set S5). Significantly enriched categories are highlighted.

A considerable proportion of metabolism-related genes showed increased transcript abundance in the QM6a background relative to their levels in the CBS999.97 background, along with enrichment in genes involved in energy-related processes and oxidative stress response (Fig. 2; Data Set S5). These genes are enriched in functions of “metabolism” (*P* = 4.35 × 10^−6^), particularly “amino acid metabolism” (*P* = 1.48 × 10^−9^), “assimilation of ammonia and metabolism of the glutamate group” (*P* = 9.64 × 10^−4^), and “metabolism of aspartate and the aspartate family,” as well as “degradation of aspartate” (*P* < 10^−5^ for both). The regulation of amino acid metabolism clearly reflects the differences in carbon source utilization preferences of CBS999.97 and QM6a as detected in the Biolog phenotype microarrays (see above). The lower growth rates of CBS999.97 on proline, l-aspartic acid, l-alanine, l-serine, and l-asparagine were all consistent with the downregulation of genes involved in metabolism of these amino acids and also, in part, their downstream pathways in CBS999.97 relative to QM6a (Data Set S5).

Moreover, enrichment was observed in genes involved in “metabolism of the pyruvate family and d-alanine” (*P* = 4.26 × 10^−4^), “purine metabolism” (*P* = 2.65 × 10^−3^), “C compound and carbohydrate metabolism” (*P* = 1.20 × 10^−3^), and, here specifically, “sugar, glucoside, polyol, and carboxylate catabolism” (*P* = 1.89 × 10^−3^), as well as “C-2 compound and organic acid metabolism” (*P* = 2.21 × 10^−3^). “Fatty acid metabolism” (*P* = 2.90 × 10^−3^), functions related to “energy” (*P* = 9.40 × 10^−8^), “respiration” (*P* = 4.09 × 10^−4^), ribosomal proteins and translation (*P* < 10^−3^), “cellular transport” (*P* = 1.55 × 10^−5^), and “oxidative stress response” (*P* = 9.64 × 10^−4^), and functions in “oxygen and radical detoxification” (*P* = 4.21 × 10^−4^) were further enriched categories among genes downregulated in CBS999.97. Genes upregulated in the CBS999.97 background showed enrichment in “extracellular metabolism and protein degradation” (*P* = 10^−3^), “translation initiation” (*P* = 4.09 × 10^−3^), and “photoperception and -response” (*P* = 3.38 × 10^−3^).

The carbon source utilization data revealed increased growth on hemicellulose and lactose degradation-associated substrates. Accordingly, genes involved in lactose degradation and that channel metabolites into the pentose phosphate pathway were differentially regulated between strains with QM6a and CBS999.97 backgrounds (Fig. S6), particularly *lxr3*, *lxr4*, *gal1*, *gal10*, and *bga1*. Moreover, *hxk1*, which is essential for growth on fructose by T. reesei (30), was strongly upregulated in QM6a, which is also consistent with the increased growth of QM6a on fructose (Fig. S3). Specifically, the transcript levels of 17 glycoside hydrolases were increased in the CBS999.97 background and 25 were decreased. The transcript levels of one of the two major cellulase genes of T. reesei, *cel6a*, were more than 15-fold decreased, along with those of several beta glucosidases of glycoside hydrolase family 3 (*cel3A*/TR_76672, *cel3C*/TR_82227, *cel3D*/TR_46816, and *cel3E*/TR_76227). In contrast, the transcript abundances of *cel3B*/TR_121735 and *bgl3i*/TR_47268 were strongly increased and the transcript level of the beta galactosidase gene *bga1* was 92-fold reduced (Data Set S5). The gene encoding the regulator BglR showed considerably higher transcript levels in CBS999.97.

Additionally, the transcript levels of three genes encoding catalases/peroxidases (*cat8*, *cat9*, and *cat10*), as well as two superoxide dismutases (*sod1* and *sod3*), were decreased in CBS999.97, suggesting an altered response to oxidative stress conditions in this strain. Also, 14 genes involved in lipid metabolism, 4 genes involved in nitrogen metabolism, and 11 genes associated with sexual development were downregulated.

### Influence of female sterility of QM6a on gene regulation.

We evaluated differential gene expression between female fertile CBS999.97 strains carrying both mating types, the two fertile lines FF1 and FF2, and the female sterile strain QM6a. We found 90 genes to be differentially regulated by more than 2-fold (*P* < 0.01). Thirty-five genes were downregulated and 55 genes upregulated in fertile strains compared to their expression in the female sterile QM6a strain (Data Set S6). Among the genes downregulated in female fertile strains, we found the catalase-encoding gene *cat7*, four genes encoding glycoside hydrolases of families 2, 30, 76, and 95 (TR_76852, TR_69736, TR_122495, and TR_111138), the sulfite oxidase gene encoding TR_76601, and two transporter genes (TR_71010 and TR_121441), as well as a putative aquaporin gene (major intrinsic protein superfamily; TR_82321). Genes upregulated in female fertile strains compared to their expression in female sterile QM6a include the GH family 5-encoding TR_53731, phospholipase C-encoding TR_111102, and the two transporter genes TR_106556 and TR_70934. Even though sexual development does not occur during growth in liquid medium with cellulose as the carbon source, the transcript abundance of the pheromone precursor gene *hpp1* was still considerably (∼14-fold) upregulated in QM6a compared to its expression in female fertile strains.

With more than 40-fold upregulation, TR_123084, a putative secreted short-chain dehydrogenase, was the most strongly differentially regulated gene. We investigated whether this gene is coregulated with genes for specific functions, which might hint at its potential physiological relevance. Therefore, we evaluated gene expression patterns in the wild type and mutants with mutations impacting photoreceptors (31) and components of the heterotrimeric G-protein pathway (32) grown on cellulose in light and darkness. The 152 coregulated genes were enriched in functions of “secondary metabolism” (*P* = 7.30 × 10^−04^), specifically, “metabolism of acetic acid derivatives, thioredoxin, glutaredoxin, or glutathione,” and moreover, in “substrate transport” (*P* = 1.58 × 10^−04^), as well as “defense and resistance.”

### Secondary metabolite profiles in QM6a and CBS999.97.

**FIG 3:**
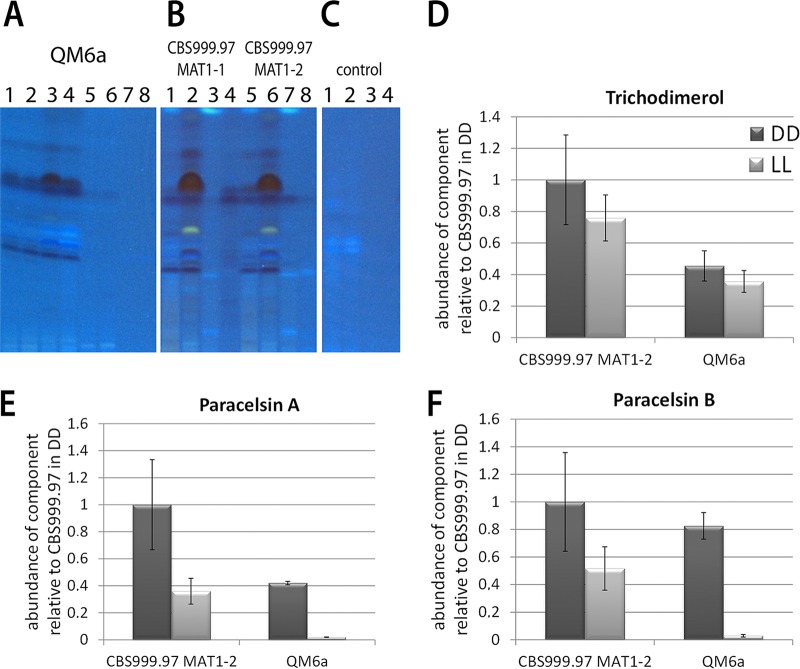
Analysis of secreted metabolites of QM6a and CBS999.97. Secreted metabolites were analyzed in minimal medium with glucose as the carbon source as follows: QM6a (A) buffered to pH 5.0 using solid (lanes 1 and 2) and liquid (lanes 3 and 4) medium or unbuffered solid (lanes 5 and 6) and liquid (lanes 7 and 8) minimal medium, CBS999.97 MAT1-1 (B) at pH 5.0 using solid (lane 1) or liquid (lane 2) medium and unbuffered solid (lane 3) or liquid (lane 4) medium, and CBS999.97 MAT1-2 1 at pH 5.0 using solid (lane 5) or liquid (lane 6) medium and unbuffered solid (lane 7) or liquid (lane 8) medium. (C) The same media without fungi were used as controls: buffered solid (lane 1) and liquid (lane 2) media and unbuffered solid (lane 3) and liquid (lane 4) media. Two biological replicates were considered in each case. High-performance thin-layer chromatography (HPTLC) plates were documented at 366 nm without derivatization. (D to F) Quantification of selected metabolites in QM6a and CBS999.97. Strains were grown on minimal medium with cellulose as the carbon source in constant light (LL) and constant darkness (DD). The abundance of each compound was related to the biomass produced and is shown relative to the result for CBS999.97 MAT1-2 in darkness. Error bars indicate standard deviations.

The finding that genes containing a high number of nonsynonymous SNPs were enriched in functions of secondary metabolism (Data Set S1) indicates that QM6a and CBS999.97 likely differ in secondary metabolite production. This conclusion is supported by the transcriptome analysis, which identified differences in the regulation of genes involved in secondary metabolism in the CBS999.97 background compared to their regulation in the QM6a background, hence suggesting altered capabilities for the production of secondary metabolites. In order to discern differences in the regulation of secondary metabolism, we analyzed the metabolites secreted from cultures grown under different conditions with high-performance thin-layer chromatography (HPTLC). In accordance with the transcriptome data, CBS999.97 showed clear differences from QM6a, especially upon growth in buffered liquid Mandels-Andreotti minimal medium with glucose as the carbon source in standing culture (Fig. 3A and B). Between CBS999.97 MAT1-1 and MAT1-2, only marginal differences were observed (Fig. 3B).

Quantitative mass spectrometry (liquid chromatography–tandem mass spectrometry [LC-MS/MS]) multitarget analysis of known fungal toxins and metabolites confirmed clear differences between QM6a and CBS999.97. Trichodimerol (Fig. 3D) and paracelsin A (Fig. 3E) were present in the medium at higher levels for CBS999.97 than for QM6a upon growth on cellulose in light and darkness (Fig. 3). The abundance of paracelsin B was strongly decreased in QM6a grown in light (Fig. 3F). The slight differences in the ratios of paracelsin A and B in QM6a and CBS999.97 were unexpected, although supplementation of different amino acids can shift the prevalence of a variant of an NRPS-associated metabolite in bacteria and fungi (33, 34). As QM6a and CBS999.97 differ in the regulation of amino acid metabolism, as outlined above, altered precursor availability may be responsible for these different ratios.

## DISCUSSION

The genus Trichoderma is one of the most widely used groups of fungi for both industrial applications in enzyme production and agricultural applications (35). The biotechnological workhorse among these fungi is Trichoderma reesei, currently one of the most frequently used filamentous fungi in industry (3). The original isolate of T. reesei, QM6a, from the Solomon Islands, is the progenitor of all strains now used in research and industry. However, numerous other isolates of T. reesei have since been obtained from diverse habitats (36).

With analysis of sexual development in T. reesei, we also found that QM6a is female sterile (12). In Magnaporthe oryzae, loss of the ability to reproduce sexually was observed after a certain number of rounds of cultivation under asexual conditions. Since this defect could not be restored by cultivation under different stress conditions, it was concluded that a mutation caused the defect. Interestingly, the female sterile mutants then released the spores they produced more efficiently, which is considered crucial for the invasion of natural populations by female sterile mutants (21). In Penicillium roqueforti, fertility also decreases during industrial use and the necessary clonal replication there (20). A similar phenomenon may have happened for QM6a, but we have isolates from natural environment that are female sterile as well.

The genes required for female fertility were reintroduced into T. reesei female sterile strains by crossing with the female fertile CBS999.97 and performing several rounds of backcrossing to regain the initial geno- and phenotype. This was first achieved for QM9414 (16) and then for QM6a (17). The phenotypes of QM6a and CBS999.97, however, differ considerably, and crosses resulted in a large number of inviable progeny and segmental aneuploidy (19). Accordingly, the progeny of crosses of CBS999.97 and QM6a display diverse phenotypes that reflect the significant differences between the strains. In general, the backcrossed strains are phenotypically almost indistinguishable from QM6a, except for a pronounced difference in sporulation efficiency in the dark (16).

The objective of this study was to investigate the differences between CBS999.97, QM6a, and backcrossed female fertile strains with the QM6a genetic background. Considerable strain-specific differences in industrially relevant strains were reported earlier; for example, for Aspergillus niger (37). While differences between CBS999.97 and QM6a might be attributable to the large geographic distance separating their original habitats, comparison with the backcrossed strains enables analysis of female fertility-related and mating type-linked features. We found considerable differences in gene regulation, secondary metabolite production, and growth on different carbon sources between the two strain backgrounds. These differences reflect a generally different physiology and not just a few specific gene mutations or genomic alterations due to horizontal gene transfer. The regulation of secondary metabolism shows significant differences in QM6a and CBS999.97, which suggests altered strategies of communication with the environment and, likely, also of defense.

For Penicillium chrysogenum, analysis of the functions of mating type loci revealed relevance in germination, surface properties of conidiospores, and light-dependent asexual sporulation (38). More importantly, the mating type locus also influences penicillin production, hyphal morphology, and conidium formation in P. chrysogenum (39). Differential gene regulation in the two mating types was also observed in Podospora anserina (27). In our study, we investigated gene regulation between mating types in two different strain backgrounds (QM6a background in FF strains and CBS999.97 background). Differences due to mating type in these T. reesei strains were found between strains in the QM6a background and between strains of different mating types in the CBS999.97 background, but only for a very few genes was differential regulation consistently detected in both backgrounds. By comparing the different strain backgrounds we avoided the identification of false-positive differences in regulation that are specific mating type-dependent differences in the QM6a background or the CBS999.97 background but not generally in T. reesei. No species-specific mating type-dependent differences in physiology can be deduced for T. reesei other than the known differences in regulation of the pheromone system (40). Our analysis clearly shows that backcrossing can be used to restore the original strain background after acquisition of female fertility from CBS999.97. QM6a and the female fertile backcrossed strains show very similar growth and gene regulation patterns compared to those of CBS999.97.

Numerous mutations have previously been shown to cause female sterility (41), in many cases without any predictable connection to sexual processes (42). Among the strains created in the N. crassa whole-genome knockout program, 58 show no production of protoperithecia (43), the female reproduction organs. The functions of the deleted genes are enriched in metabolism, including C compound and carbohydrate metabolism, but also secondary metabolism (data not shown). For T. reesei, with respect to female fertility, several glycoside hydrolase gene transporters are among the genes differentially regulated between female fertile strains and female sterile QM6a. However, for none of these genes has a major influence on cellulase gene expression been shown so far. Nevertheless, the finding of a connection between female fertility and regulation of metabolism-related genes is in line with earlier findings.

Considering sexual crossing as a promising tool, it has to be kept in mind that all the strains currently used in research and the applied production strains derived from QM6a are female sterile. Crossing any of these strains with a female fertile partner, whether a wild type or a backcrossed strain, will yield female sterile strains among the progeny. Hence, a difference in performance for a given target protein or in the efficiency of the produced enzyme mixture due to female fertility or sterility cannot be excluded. Nevertheless, extensive analysis of progeny from production strains will be required to unequivocally confirm an influence of fertility on performance to confirm this correlation.

In summary, we found that although CBS999.97 and QM6a can undergo sexual crossing, these strains exhibit considerable phenotypic differences in growth on different carbon sources, gene regulation, and secondary metabolism. Transcriptome analysis suggested additional differences both in dealing with oxidative stress and in energy-related functions. Despite these differences, backcrossing of QM6a-derived strains to acquire sexual competence successfully restores the majority of these regulatory differences, albeit some alterations due to mating type or female fertility remain. Hence, our study provides a perspective for knowledge-based strain improvement by sexual development in T. reesei: from progeny of crosses, strains of both mating types should be included in screenings, as well as female fertile and female sterile strains, in order to increase the probability of obtaining strains with the desired phenotypes.

## MATERIALS AND METHODS

### Microbial strains and cultivation conditions.

The T. reesei wild-type strain QM6a (ATCC 13631) and strains CBS999.97 MAT1-1, CBS999.97 MAT1-2 (12), FF1, and FF2 (17), as well as sister strains from different crossing lines of FF1 and FF2, FF1a, -b, and -c and FF2a, -b, and -c (Fig. 1A), were used throughout this study. FF1 and FF2 are female fertile derivatives of QM6a, which was crossed with CBS999.97 MAT1-1 and thereafter backcrossed 10 times to acquire sexual competence while retaining the QM6a strain background (17). Propagation of strains was done on malt extract medium. For inoculum preparation, strains were grown in constant darkness for 10 days to avoid any influence of circadian rhythms or random light pulses on our cultures.

For RNA analysis, strains were grown in Mandels-Andreotti minimal medium (44) supplemented with 0.1% (wt/vol) peptone at 200 rpm and 28°C in a rotary shaker in constant darkness. Microcrystalline cellulose at 1% (wt/vol) was used as a carbon source. Mycelia were harvested using a red safety light (Philips PF712E darkroom lamp) in order to avoid the influence of a light pulse on transcript levels.

### Sequencing and assembly of the genome of CBS999.97.

Strain CBS999.97 was sequenced with both 454 and Illumina technology (19), and paired-end Illumina sequencing was performed at the Joint Genome Institute (JGI; Walnut Creek, CA, USA). The Illumina and 454 reads were combined into a single assembly using the Newbler runAssembly command (45) and scaffolded using MeDuSa (46) based on the consensus order and orientation of T. reesei version 2, T. atroviride version 2, and T. virens version 2 genomes downloaded from JGI. Data are available under NCBI BioProject accession number PRJNA348760 and GenBank accession number MLIW00000000. The Illumina reads from CBS999.97 were mapped to the QM6a genome using Bowtie (47) to identify SNPs, which were annotated based on the QM6a reference annotation (8).

### Biolog phenotype microarray analysis.

The growth of T. reesei strains was analyzed essentially as described previously (48, 49) using the Biolog system for 96 carbon sources. For QM6a, CBS999.97 MAT1-1, and CBS999.97 MAT1-2, biological triplicates were used, and for FF1 and FF2, three sister strains from individual crossing lines were used as replicates (FF1a, -b, and -c and FF2a, -b, and -c) to rule out single recombination effects in one strain. Strains were grown for 72 h in constant darkness as clear differences in growth patterns are apparent for T. reesei after this time, while the onset of conidiation occurs only after 72 h and, hence, does not interfere with the results (49).

### Transcriptome analysis.

For transcriptome analysis, QM6a, CBS999.97 MAT1-1, and CBS999.97 MAT1-2 were used. In the case of the backcrossed strains with the QM6a background, we used FF1a and FF1b, as well as FF2a and FF2b, as replicates to increase the robustness of our study and to exclude strain-specific features. Strains were grown in parallel and harvested as described above. Total RNA was isolated, and RNA quality was checked by using a Bio-Rad Experion system as described previously (50); only high-quality samples with RNA integrity numbers (RINs) of ≥9 were used for further analyses (50). For microarray experiments, we used the full-service gene expression analysis of Roche NimbleGen (Madison, WI, USA). This service features standardized single-channel hybridization of slides, and hence, batch-to-batch comparison of gene expression values is enabled. For CBS999.97, custom arrays in the 4-by-72,000 format were designed based on the genome data obtained in this study. Gene and protein models of CBS999.97 were correlated with those of QM6a by bidirectional best-hit analysis to enable comparability. Data are available at Gene Expression Omnibus under accession number GSE89103.

### Bioinformatic analyses.

Bioinformatic analysis of transcriptome data was performed with Partek Genomics Suite 6.5 (Partek, Inc., St. Louis, MO, USA), which uses analysis of variation (ANOVA) to identify statistically significant differential regulation of genes. ANOVA enables reliable comparison of more than two samples by avoiding type I errors (“a positive assumption is false”). For the analysis in this study, the individual data sets were combined according to the respective research question and treated as replicates (for example, all data sets for MAT1-1 strains versus all data sets for MAT1-2 strains). For gene expression pattern analyses and hierarchical clustering, the open source software HCE3.5 (51) was used. Functional category analysis (FunCat) was done using the online resource at MIPS with the latest version of May 2014 (http://mips.helmholtz-muenchen.de/funcatDB/) (52) The *P* values shown indicate significant enrichment of a gene group in the respective group of regulated genes. For analysis of genomic clusters, the genomic locations of reciprocal best blast hits between QM6a and CBS999.97 were determined and CAZyme clusters, as well as induced clusters, were compared to those identified in QM6a (53).

### Secondary metabolite analysis.

For analysis of secondary metabolite patterns, QM6a and CBS999.97 were grown on agar plates or in standing cultures in petri dishes with Mandels-Andreotti minimal medium with 1% (wt/vol) glucose as the carbon source, either buffered at pH 5.0 with 0.1 M phosphate-citrate buffer or unbuffered. Agar pieces or liquid medium from at least three plates were pooled, and two independent biological replicates were evaluated. High-performance thin-layer chromatography (HPTLC) was performed and the results were visualized as described previously (17), except that separation was done with chloroform and 1 mM trifluoroacetic acid in methanol. Detection and quantification of selected fungal secondary metabolites was performed as described previously (54), using a QTrap 5500 MS/MS system (Applied Biosystems, Foster City, CA) equipped with a TurboIonSpray electrospray ionization (ESI) source and a 1290 series ultra-high-performance liquid chromatography (UHPLC) system (Agilent Technologies, Waldbronn, Germany). Chromatographic separation was performed at 25°C on a Gemini C_18_ column (150 mm long, 4.6-mm inner diameter [i.d.], and 5-μm particle size) equipped with a C_18_ SecurityGuard cartridge (4 mm long and 3-mm i.d.) (all from Phenomenex, Torrance, CA, USA). Calibration was performed with a serial dilution of a multianalyte stock solution. Using this approach, routine detection and quantification of 710 metabolites was performed (M. Sulyok, D. Stadler, D. Steiner, M. Piatkowska, F. Berthiller, and R. Krska, unpublished data).

### Accession number(s).

Data for the CBS999.97 genome are available under NCBI BioProject accession number PRJNA348760 and GenBank accession number MLIW00000000. Transcriptome data are available at Gene Expression Omnibus under accession number GSE89103.

## Supplementary Material

Supplemental material
