# Supplementary material for: Omics Analyses of Trichoderma reesei CBS999.97 and QM6a Indicate the Relevance of Female Fertility to Carbohydrate-Active Enzyme and Transporter Levels
Source: Appl Environ Microbiol. 2017 Oct 31;83(22):e01578-17. doi: 10.1128/AEM.01578-17 (PMC5666144; doi:10.1128/AEM.01578-17)
Supplement: Supplemental material [file supp_83_22_e01578-17__index.html]

Supplemental material 

# Omics Analyses of Trichoderma reesei CBS999.97 and QM6a Indicate the Relevance of Female Fertility to Carbohydrate-Active Enzyme and Transporter Levels

## Supplemental material

- Supplemental file 1 -

  Conservation of gene clusters (Note S1); distances between genes in clusters (Fig. S1); mating type-dependent growth characteristics (Fig. S2); growth characteristics of strains with different genetic backgrounds (Fig. S3); growth characteristics as influenced by female fertility (Fig. S4); regulation patterns of transcription factors (Note S2); regulation patterns of transcription factor-encoding genes in different genetic backgrounds (Fig. S5); differential regulation of pentose phosphate pathway and glycolysis (Fig. S6); gene models retaining SNPs from CBS999.97 in all three tested female fertile backcrossed strains (Table S1); results of progeny analysis for the presence of selected genes from all three genomic regions which had been retained in all three female fertile strains (Table S2).

  PDF, 1.5M
- Supplemental file 2 -

  Analysis of SNP occurrence between the reference genome of QM6a and CBS999.97 (Data Set S1).

  XLSX, 854K
- Supplemental file 3 -

  Comparison of CAZyme clusters between QM6a and CBS999.97 (Data Set S2).

  XLSX, 93K
- Supplemental file 4 -

  Distribution of CAZyme-encoding genes according to their regulation patterns (Data Set S3).

  XLSX, 71K
- Supplemental file 5 -

  Gene regulation patterns of transcription factor-encoding genes (Data Set S4).

  XLSX, 27K
- Supplemental file 6 -

  Gene regulation differences in the QM6a and CBS999.97 genetic backgrounds (Data Set S5).

  XLSX, 254K
- Supplemental file 7 -

  Gene regulation differences in female fertile and female sterile strain backgrounds (Data Set S6).

  XLS, 119K
