# Supplementary material for: Omics Analyses of Trichoderma reesei CBS999.97 and QM6a Indicate the Relevance of Female Fertility to Carbohydrate-Active Enzyme and Transporter Levels
Source: Appl Environ Microbiol. 2017 Oct 31;83(22):e01578-17. doi: 10.1128/AEM.01578-17 (PMC5666144; doi:10.1128/AEM.01578-17)
Supplement: Supplemental material [file AEM.01578-17_zam999118153s1.pdf]

# Equal and different –genome, transcriptome, metabolic capacities and secondary metabolism of *Trichoderma reesei* CBS999.97 indicate high physiological variability in this species

Doris Tisch[1], Kyle R. Pomraning[2,3], James R. Collett[2], Michael Freitag[3], Scott E. Baker[2], Chia-Ling Chen[4], Paul Wei-Che Hsu[4], Yu Chien Chuang[4], Andre Schuster[1], Christoph Dattenböck[5], Eva Stappler[5], Michael Sulyok[6], Stefan Böhmdorfer[7], Josua Oberlerchner[7], Ting-Fang Wang[4] and Monika Schmoll[1,5]*

# Supplementary Material

**Table of contents**

Supplementary note 1 - Conservation of gene clusters 2

Figure S1 Distances in clusters 3
Figure S2 Mating type dependent growth characteristics 4
Figure S3 Growth characteristics of strains with different genetic background 5
Figure S4 Growth characteristics as influenced by female fertility 6

Supplementary note 2 - Regulation patterns of transcription factors 7

Figure S5 Regulation patterns of transcription factor encoding genes 8
Figure S6 Differential regulation pentose phosphate pathway and glycolysis 9

References 10

Supplementary table S1 11
Supplementary table S2 23

*Supplementary note 1 - Conservation of gene clusters between QM6a and CBS999.97*

In contrast to other Sordariomycetes, in *T. reesei* a high proportion of CAZyme encoding genes are distributed non-randomly in the genome and form clusters that are located between regions of synteny with related fungi (1). This clustering is assumed to be relevant for efficient CAZyme production in *T. reesei* and beneficial effects due to common promotor areas cannot be excluded.
We were therefore interested whether these CAZyme gene clusters from QM6a were conserved in CBS999.97 or if alterations had taken place. Therefore we repeated the identification of CAZyme clusters with parameters that not only validated previously identified clusters (1) but also revealed additional new clusters. (Stappler et al., manuscript submitted); these analyses were used as basis for comparison with gene order and distances in CBS999.97 (Supplementary file 4). Generally, the distances between the genes in these clusters was largely similar in CBS999.97 and QM6a (Supplementary file 4, Figure S1). Interestingly, we found alterations in several clusters. Cluster 2 comprises several predicted alpha- and beta glycosidases of different glycoside hydrolase (GH) families as well as the D-mannitol dehydrogenase encoding *lxr1* (2) and the xylanase encoding *xyn1*. This cluster overlaps with a light- and ENV1- regulated cluster (3). The cluster appears divided after *xyn1* in CBS999.97. However, checking with the recent high-quality assembly provided by (4), we found that in QM6a a misassembly has happened at this locus and that cluster 2 is indeed two clusters on different chromosomes (chromosome 5 and 7)..
Cluster 14 comprises two carbohydrate esterases and two glycoside hydrolases. It is located on scaffold 7 in QM6a is split between two scaffolds in CBS999.97, with distances to scaffold borders that are too large to indicate conservation of synteny, as they are also larger than the distances in QM6a. In this case the new assembly confirms the QM6a assembly. From cluster 35, which is confirmed in the new assembly and includes genes encoding enzymes of carbohydrate binding module (CBM) family 13 as well as glycoside hydrolases and one carbohydrate esters, the chitinase encoding *chi18-16* is missing in the middle of this cluster on scaffold 13. Instead, it is found on scaffold 58, indicating that also cluster is not fully conserved.
Cluster 37 comprising the N-acetyl-ß-D-glucosaminidase gene *nag2* (5), the cellobiohydrolase gene *cbh1* (6) and the xylanase *xyn4* (7), also appears divided in CBS999.97, albeit considering the lengths of the respective scaffolds, the genes are located close to the borders and synteny of cluster 37 is likely to be conserved.
Consequently, most of the CAZyme clusters detected in QM6a are conserved in CBS999.97. Cluster 2 is likely to be actually somewhat shorter and ending with *xyn1*, while the differences between CBS999.97 and QM6a for cluster 14 and cluster 35 may indicate that their gene order might not be functionally relevant.

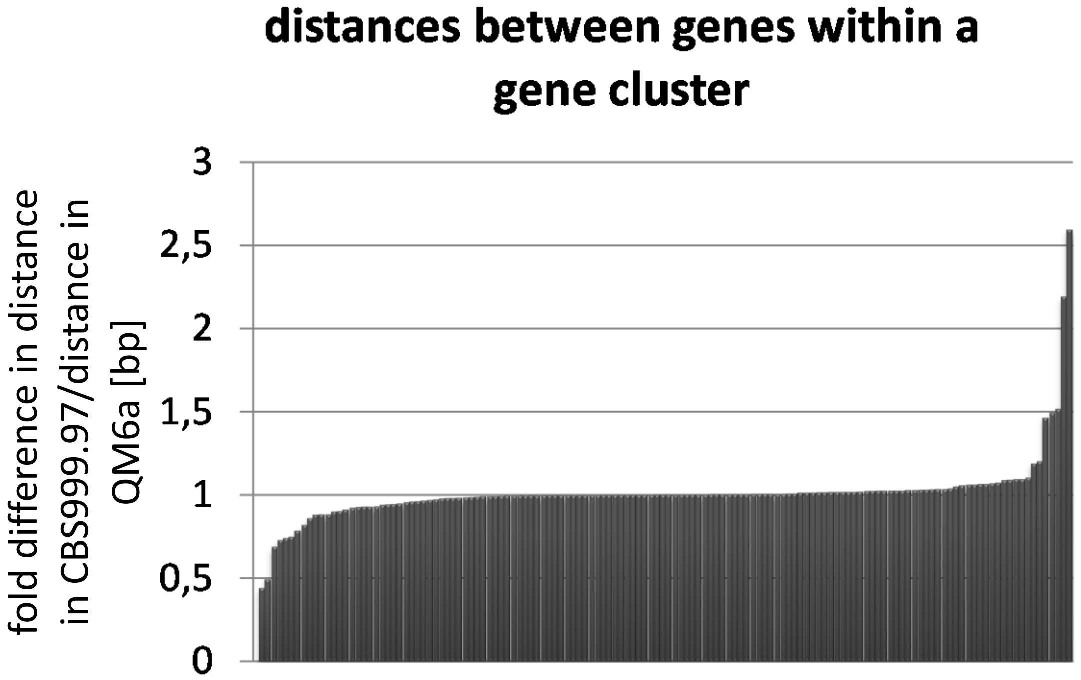


Figure S1. **Distances between genes within a cluster in CBS999.97 compared to distances between the same two genes in QM6a**

Figure S2

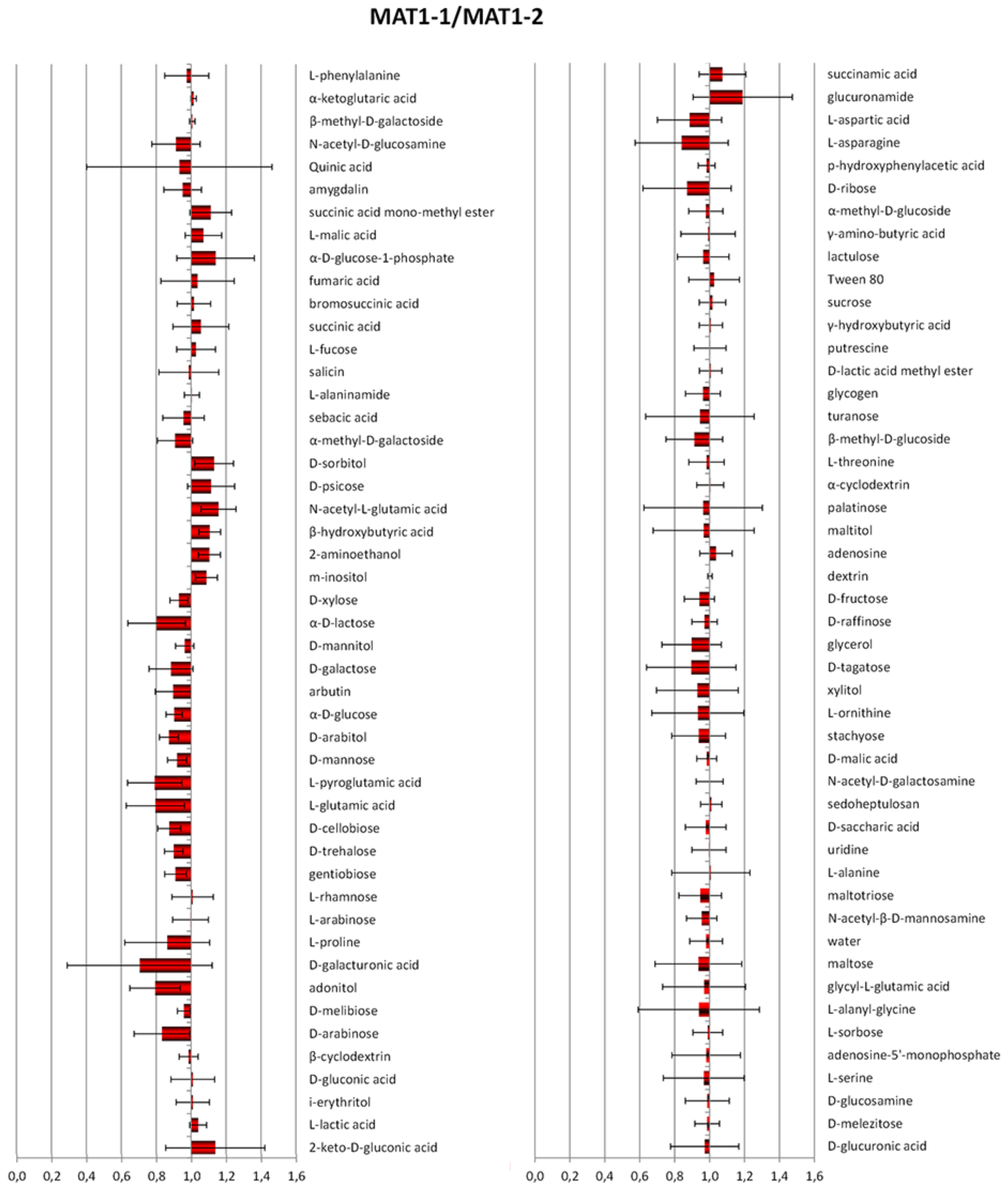


Figure S2. **Mating type dependent growth characteristics**.
Growth was analyzed on 95 carbon sources after 72 hours in QM6a (MAT1-2), FF1 a, b, and c (female fertile sister strains of mating type MAT1-1) and FF2 a, b, and c (female fertile sister strains of mating type MAT1-2). All MAT1-1 strains were treated as replicates versus MAT1-2 strains. Error bars show standard deviation. Values above 1 indicate better growth of MAT1-1 strains, while values below 1 show that this carbon sources supported better growth in MAT1-2 strains.

Figure S3

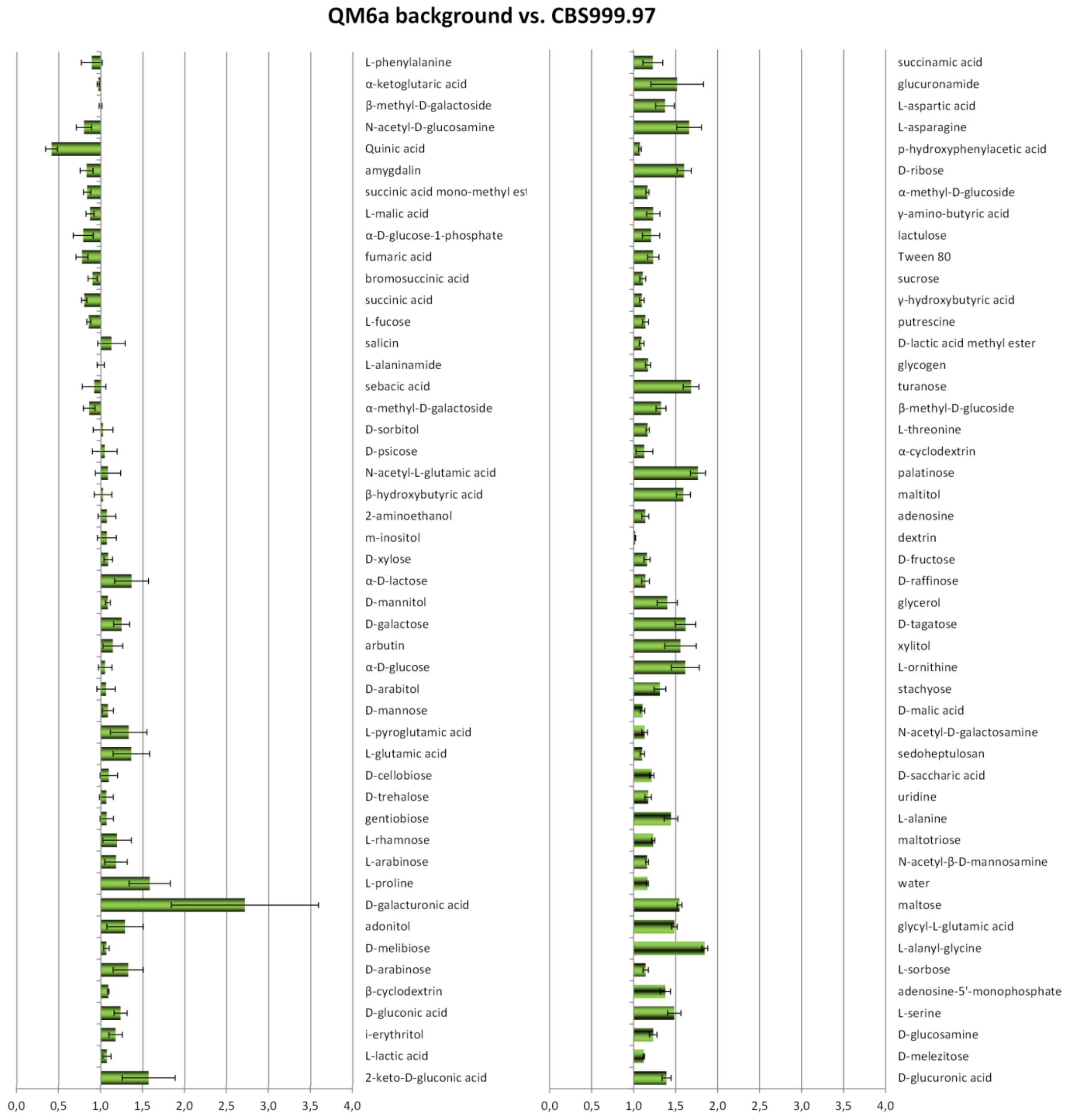


Figure S3. **Growth characteristics of strains with different genetic background**.
Growth was analyzed on 95 carbon sources after 72 hours in QM6a (MAT1-2), FF1 a, b, and c (female fertile sister strains of mating type MAT1-1) and FF2 a, b, and c (female fertile sister strains of mating type MAT1-2). All strains with QM6a background (QM6a, FF1 and FF2 strains) were treated as replicates versus CBS999.97 derived strains. Error bars show standard deviation.
Values above 1 indicate better growth of QM6a, while values below 1 show that this carbon sources supported better growth in CBS999.97 than in QM6a.

Figure S4

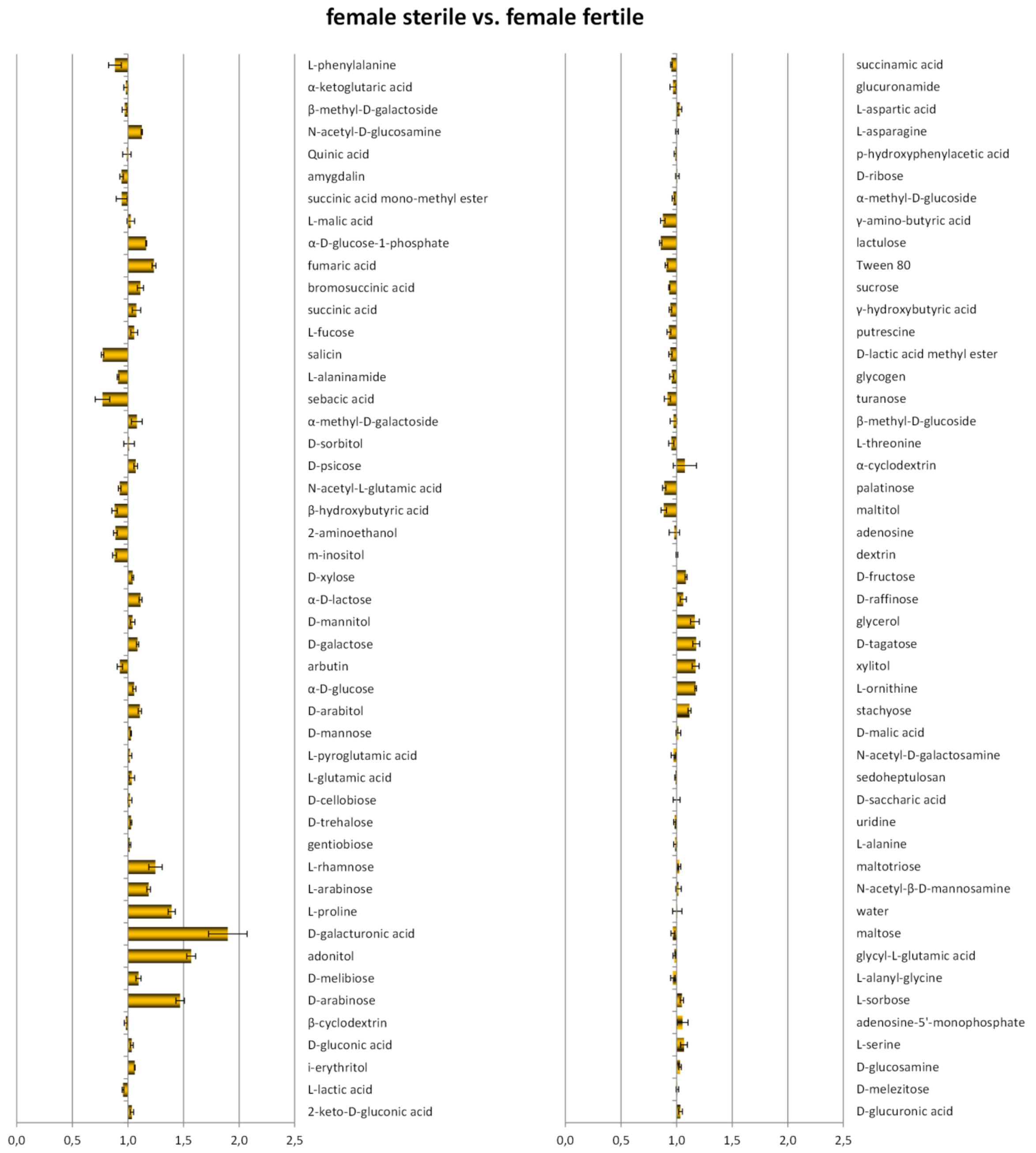


Figure S4. **Growth characteristics as influenced by female fertility**.
Growth was analyzed on 95 carbon sources after 72 hours in QM6a (MAT1-2), FF1 a, b, and c (female fertile sister strains of mating type MAT1-1) and FF2 a, b, and c (female fertile sister strains of mating type MAT1-2). All female fertile strains (CBS999.97, FF1, FF2) were treated as replicates versus female sterile QM6a. Error bars show standard deviations. Values above 1 indicate better growth of female sterile strains, while values below 1 show that this carbon sources supported better growth in female fertile strains.

*Supplementary note 2 - Regulation patterns of transcription factors*

Analysis of regulation of transcription factor genes upon growth on cellulose showed altered patterns in QM6a background versus CBS999.97 background (Figure S5), while only few differences between QM6a and the female fertile derivatives FF1 and FF2 were detected. Specifically, TR_103158, TR_102497, TR_122253 and TR_102499 are significantly upregulated in FF1 and FF2 strains. Overall, hierarchical clustering revealed four discete clusters (Supplementary file 6).

Transcription factor genes of cluster 1 (upregulation in QM6a background) are enriched in "regulation of amino acid metabolism" (*p* value 1.51 x $10^{-07}$), "regulation of nitrogen, sulfur and selenium metabolism" (*p* value 8.52 x $10^{-17}$), "regulation of C-compound and carbohydrate metabolism" (*p* value 2.60 x $10^{-07}$), "secondary metabolism" (*p* value 4.08 x $10^{-04}$) and functions of "development" (*p* value 6.58 x $10^{-04}$). This cluster includes the known cellulase regulators *ace2*, *ace3* and *xyr1*, the developmental regulator *ste12*, and the regulators of nitrogen metabolism *nirA*, *nit2* and *nit4* (Figure S5). For cluster 4 (down-regulation in QM6a background) we found an enrichment in the same categories, indicating that different transcription factors are used for these functions in CBS999.97 and QM6a background upon growth on cellulose. Only the enrichment in functions of development was specific to cluster 1. Cluster 4 includes the cellulase regulators *bglR* and *ace1*, a regulator involved in ascus development, *asd4*, and the regulator involved in unfolded protein response, *hac1*. Clusters 2 and 3 comprise only few genes with inconsistent regulation in strain backgrounds.

Figure S5

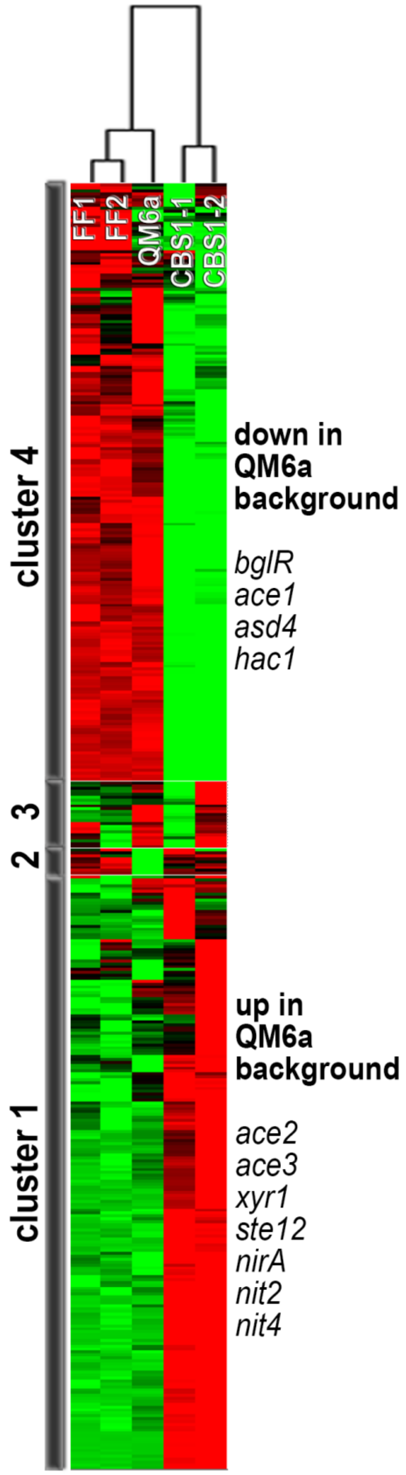


Figure S5. **Regulation patterns of transcription factor encoding genes in different genetic backgrounds**

Transcript levels of transcription factor encoding genes from CBS999.97 in both mating types, QM6a and FF1 and FF2 strains were analyzed by hierarchical clustering. Transcription factor encoding genes upregulated in strains with QM6a background (QM6a, FF1 and FF2 strains) are shown with cluster 1, those downregulated in QM6a background strains compared to CBS999.97 in both mating types are shown with cluster 1.

Figure S6

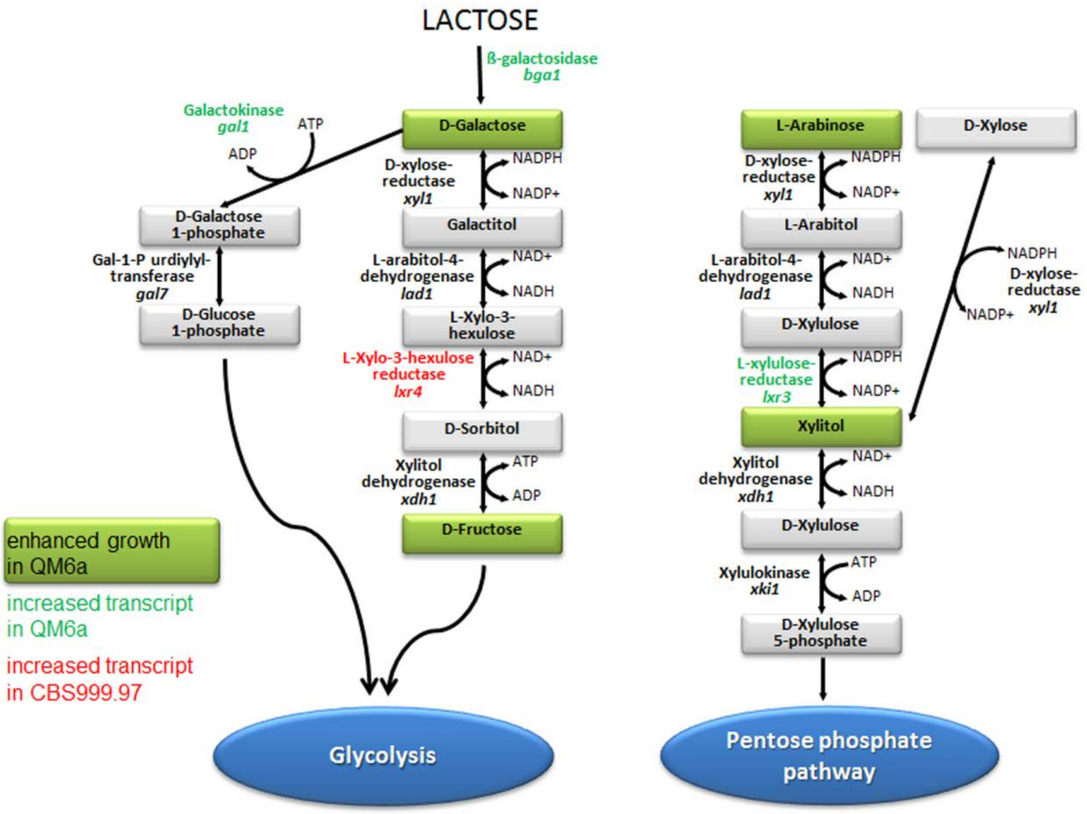


Figure S6 **Differential regulation of growth and transcription associated upstream of the pentose phosphate pathway and glycolysis.**
Genes encoding enzymes involved in these pathways are given in green if upregulated in QM6a background and in red if upregulated in the CBS999.97 genetic background. Pathway intermediates are highlighted in green if the BIOLOG phenotype microarray showed enhanced growth of QM6a related strains on this carbon source versus CBS999.97 strains.

REFERENCES


1. **Martinez D, Berka RM, Henrissat B, Saloheimo M, Arvas M, Baker SE, Chapman J, Chertkov O, Coutinho PM, Cullen D, Danchin EG, Grigoriev IV, Harris P, Jackson M, Kubicek CP, Han CS, Ho I, Larrondo LF, de Leon AL, Magnuson JK, Merino S, Misra M, Nelson B, Putnam N, Robbertse B, Salamov AA, Schmoll M, Terry A, Thayer N, Westerholm-Parvinen A, Schoch CL, Yao J, Barbote R, Nelson MA, Detter C, Bruce D, Kuske CR, Xie G, Richardson P, Rokhsar DS, Lucas SM, Rubin EM, Dunn-Coleman N, Ward M, Brettin TS.** 2008. Genome sequencing and analysis of the biomass-degrading fungus *Trichoderma reesei* (syn. *Hypocrea jecorina*). Nat Biotechnol **26:**553-560.
2. **Metz B, de Vries RP, Polak S, Seidl V, Seiboth B.** 2009. The *Hypocrea jecorina* (syn. *Trichoderma reesei*) *lxr1* gene encodes a D-mannitol dehydrogenase and is not involved in L-arabinose catabolism. FEBS Lett **583:**1309-1313.
3. **Tisch D, Schmoll M.** 2013. Targets of light signalling in *Trichoderma reesei*. BMC Genomics **14:**657.
4. **Marie-Nelly H, Marbouty M, Cournac A, Flot JF, Liti G, Parodi DP, Syan S, Guillen N, Margeot A, Zimmer C, Koszul R.** 2014. High-quality genome (re)assembly using chromosomal contact data. Nat Commun **5:**5695.
5. **Lopez-Mondejar R, Catalano V, Kubicek CP, Seidl V.** 2009. The beta-N-acetylglucosaminidases NAG1 and NAG2 are essential for growth of *Trichoderma atroviride* on chitin. FEBS J **276:**5137-5148.
6. **Seiboth B, Hakola S, Mach RL, Suominen PL, Kubicek CP.** 1997. Role of four major cellulases in triggering of cellulase gene expression by cellulose in *Trichoderma reesei*. J Bacteriol **179:**5318-5320.
7. **Tenkanen M, Vrsanska M, Siika-aho M, Wong DW, Puchart V, Penttila M, Saloheimo M, Biely P.** 2013. Xylanase XYN IV from *Trichoderma reesei* showing exo- and endo-xylanase activity. FEBS J **280:**285-301.

Supplementary table 1

**Gene models retaining SNPs from CBS999.97 in all three tested female fertile backcrossed strains (FF1-3)**

| gene | contig | position | QM6a ref base | CBS mut base | QM6a ref acid | CBS mut acid | motif | gene |
|---|---|---|---|---|---|---|---|---|
| gene_id "e_gw1.2.657.1" - | 2 | 6454 | G | T | Q | K | QPQEP | |
| gene_id "e_gw1.2.657.1" - | 2 | 6483 | T | C | Q | R | PPQPE | |
| gene_id "e_gw1.2.657.1" - | 2 | 7013 | G | T | D | E | EPDVP | |
| gene_id "e_gw1.2.657.1" - | 2 | 8749 | A | G | S | P | VESKE | |
| gene_id "estExt_fgenesh5_pg.C_20006" + | 2 | 18670 | G | C | E | D | YDEDA | |
| gene_id "estExt_fgenesh5_pg.C_20006" + | 2 | 19041 | C | T | L | F | RSLYC | |
| gene_id "estExt_fgenesh5_pg.C_20006" + | 2 | 19504 | A | G | E | G | EEEEE | |
| gene_id "kg2.C_2000004" - | 2 | 24299 | T | A | T | S | SVTVQ | |
| gene_id "kg2.C_2000004" - | 2 | 24310 | T | G | Q | P | SSQPS | |
| gene_id "kg2.C_2000004" - | 2 | 24314 | A | C | S | A | PSSQP | |
| gene_id "kg2.C_2000004" - | 2 | 24531 | G | A | S | F | MVSVR | |
| gene_id "kg2.C_2000004" - | 2 | 24805 | C | T | D | N | RGDDQ | |
| gene_id "fgenesh5_pg.C_2000009" - | 2 | 28082 | C | T | D | N | LLDDD | |
| gene_id "estExt_fgenesh1_pm.C_20005" + | 2 | 44248 | A | C | I | L | YLIGG | |
| gene_id "estExt_fgenesh1_pm.C_20005" + | 2 | 44337 | G | C | K | N | DLKDG | |
| gene_id "estExt_fgenesh1_pm.C_20005" + | 2 | 44548 | A | G | I | V | SAIST | |
| gene_id "estExt_fgenesh1_pm.C_20005" + | 2 | 44550 | C | G | I | M | SAIST | |
| gene_id "estExt_fgenesh1_pm.C_20005" + | 2 | 44800 | G | A | V | M | NAVGG | |
| gene_id "estExt_fgenesh1_pm.C_20005" + | 2 | 44836 | C | A | L | I | VALGA | |
| gene_id "fgenesh5_pg.C_2000011" + | 2 | 46897 | T | G | H | Q | IMHFS | |
| gene_id "fgenesh5_pg.C_2000011" + | 2 | 46922 | G | T | V | F | LAVAT | |
| gene_id "estExt_GeneWisePlus.C_20014" + | 2 | 48184 | C | A | Q | K | KAQAA | |
| gene_id "estExt_GeneWisePlus.C_20014" + | 2 | 48323 | A | G | Q | R | YPQIE | |
| gene_id "estExt_GeneWisePlus.C_20014" + | 2 | 48465 | T | C | Y | H | AFYND | |
| gene_id "estExt_GeneWisePlus.C_20014" + | 2 | 48527 | C | A | D | E | STDTK | |

| | | | | | | |
|---|---|---|---|---|---|---|
| gene_id "estExt_GeneWisePlus.C_20014" + | 2 | 48529 | C | A | T | K | TDTKV |
| gene_id "estExt_GeneWisePlus.C_20014" + | 2 | 48791 | G | A | G | S | PPGIT |
| gene_id "estExt_GeneWisePlus.C_20014" + | 2 | 48824 | T | C | S | P | KCSVQ |
| gene_id "e_gw1.2.839.1" - | 2 | 50558 | C | A | A | S | ERAEG |
| gene_id "e_gw1.2.842.1" + | 2 | 57019 | A | G | I | M | SQIVG |
| gene_id "e_gw1.2.842.1" + | 2 | 57841 | T | C | S | P | DMSII |
| gene_id "e_gw1.2.842.1" + | 2 | 58115 | T | C | I | T | PRIRP |
| gene_id "e_gw1.2.842.1" + | 2 | 58646 | T | A | F | Y | MLFRY |
| gene_id "estExt_fgenesh5_pg.C_20019" - | 2 | 63080 | A | C | S | A | ASSSS |
| gene_id "estExt_fgenesh1_pm.C_20009" + | 2 | 84365 | G | A | V | I | NLVTL |
| gene_id "estExt_fgenesh1_pm.C_20009" + | 2 | 84677 | A | G | T | A | LPTFA |
| gene_id "estExt_fgenesh1_pm.C_20009" + | 2 | 84947 | A | G | I | V | VPIRR |
| gene_id "estExt_fgenesh1_pm.C_20009" + | 2 | 85716 | G | A | D | N | GRDSP |
| gene_id "fgenesh5_pg.C_2000025" + | 2 | 88696 | C | A | D | E | RLDTG |
| gene_id "fgenesh5_pg.C_2000025" + | 2 | 89140 | C | A | D | E | MPDSR |
| gene_id "fgenesh5_pg.C_2000025" + | 2 | 89408 | C | T | P | S | YRPAA |
| gene_id "fgenesh5_pg.C_2000025" + | 2 | 89442 | A | T | Q | L | TPQAM |
| gene_id "fgenesh5_pg.C_2000025" + | 2 | 89642 | G | A | D | N | SCDTP |
| gene_id "fgenesh5_pg.C_2000026" + | 2 | 92109 | G | T | G | V | GLGNV |
| gene_id "e_gw1.2.940.1" + | 2 | 94927 | C | T | A | V | GVADS |
| gene_id "e_gw1.2.940.1" + | 2 | 94957 | A | C | N | T | LANYG |
| gene_id "fgenesh5_pg.C_2000051" + | 2 | 166360 | C | A | N | K | PHNWV |
| gene_id "fgenesh5_pg.C_2000051" + | 2 | 166388 | A | C | N | H | VDNTS |
| gene_id "estExt_fgenesh5_pg.C_20052" - | 2 | 171738 | C | T | A | T | DDAAV |
| gene_id "e_gw1.2.965.1" + | 2 | 174603 | G | A | R | K | GARKL |
| gene_id "e_gw1.2.1065.1" + | 2 | 190832 | T | A | N | K | LENDT |
| gene_id "e_gw1.2.1065.1" + | 2 | 190977 | T | A | S | T | RYSKG |
| gene_id "fgenesh5_pg.C_2000058" - | 2 | 192073 | T | G | R | S | HRRVP |
| gene_id "fgenesh5_pg.C_2000058" - | 2 | 192211 | C | T | M | I | GVMKP |
| gene_id "fgenesh5_pg.C_2000058" - | 2 | 192282 | T | C | I | V | YKITE |

| | | | | | | | |
|---|---|---|---|---|---|---|---|
| gene_id "fgenesh5_pg.C_2000058" - | 2 | 194285 | A | T | F | I | TEFGR |
| gene_id "fgenesh5_pg.C_2000058" - | 2 | 194491 | T | A | K | M | DPKDL |
| gene_id "fgenesh5_pg.C_2000058" - | 2 | 194786 | G | A | R | C | QVRDD |
| gene_id "fgenesh5_pg.C_2000058" - | 2 | 195098 | T | C | I | V | VFIFP |
| gene_id "fgenesh5_pg.C_2000058" - | 2 | 195119 | G | C | Q | E | AQQDG |
| gene_id "e_gw1.2.845.1" - | 2 | 197048 | G | A | A | V | TKAGV |
| gene_id "e_gw1.2.245.1" + | 2 | 205123 | C | T | L | F | EILAI |
| gene_id "e_gw1.2.245.1" + | 2 | 205216 | C | G | H | D | QRHDS |
| gene_id "e_gw1.2.245.1" + | 2 | 205271 | A | G | K | R | PFKVK |
| gene_id "e_gw1.2.245.1" + | 2 | 205664 | G | A | A | T | SLASL |
| gene_id "e_gw1.2.245.1" + | 2 | 205778 | G | C | A | P | MKAVA |
| gene_id "e_gw1.2.245.1" + | 2 | 205841 | G | A | D | N | ATDVE |
| gene_id "e_gw1.2.245.1" + | 2 | 205842 | A | G | D | G | ATDVE |
| gene_id "e_gw1.2.245.1" + | 2 | 205853 | G | A | A | T | EFAFS |
| gene_id "e_gw1.2.245.1" + | 2 | 205881 | A | G | H | R | QAHSE |
| gene_id "e_gw1.2.245.1" + | 2 | 205983 | C | A | A | D | QFAVR |
| gene_id "e_gw1.2.245.1" + | 2 | 206081 | G | A | D | N | GSDSN |
| gene_id "e_gw1.2.245.1" + | 2 | 206186 | A | G | T | A | LLTDR |
| gene_id "estExt_Genewise1.C_20113" + | 2 | 221627 | T | C | L | P | GRLNM |
| gene_id "estExt_Genewise1.C_20113" + | 2 | 221650 | C | T | P | S | GGPGP |
| gene_id "estExt_Genewise1.C_20113" + | 2 | 222078 | C | T | P | S | RPPPN |
| gene_id "fgenesh5_pg.C_2000070" + | 2 | 230551 | C | A | A | D | RHAEE |
| gene_id "fgenesh5_pg.C_2000070" + | 2 | 230559 | A | G | N | D | EENLA |
| gene_id "fgenesh5_pg.C_2000070" + | 2 | 230580 | A | G | T | A | EPTPA |
| gene_id "fgenesh5_pg.C_2000070" + | 2 | 230908 | A | G | I | V | LDIGV |
| gene_id "fgenesh5_pg.C_2000070" + | 2 | 230923 | G | A | V | M | DDVYM |
| gene_id "fgenesh5_pg.C_2000070" + | 2 | 231317 | T | C | M | T | TVMLT |
| gene_id "fgenesh5_pg.C_2000070" + | 2 | 232052 | C | A | A | E | RAASL |
| gene_id "fgenesh5_pg.C_2000070" + | 2 | 232339 | G | A | V | I | TPVKT |
| gene_id "fgenesh5_pg.C_2000070" + | 2 | 232570 | T | G | Y | D | FRYCP |

| | | | | | | | |
|---|---|---|---|---|---|---|---|
| gene_id "estExt_GeneWisePlus.C_20120" - | 2 | 239262 | T | C | N | S | EWNVF |
| gene_id "fgenesh5_pg.C_2000075" - | 2 | 243100 | G | A | A | V | KEATH |
| gene_id "fgenesh5_pg.C_2000075" - | 2 | 243106 | T | C | K | R | EWKEA |
| gene_id "e_gw1.2.762.1" + | 2 | 255400 | A | G | I | M | PMIPN |
| gene_id "estExt_fgenesh1_pm.C_20026" + | 2 | 274309 | A | T | T | S | VETTL |
| gene_id "estExt_fgenesh1_pm.C_20026" + | 2 | 274706 | A | T | H | L | YGHRE |
| gene_id "e_gw1.2.991.1" - | 2 | 278881 | C | T | R | K | LTRIH |
| gene_id "kg2.C_2000021" - | 2 | 285534 | T | A | E | V | MTEKE |
| gene_id "kg2.C_2000021" - | 2 | 286535 | A | C | D | E | LYDMQ |
| gene_id "kg2.C_2000021" - | 2 | 286740 | G | A | T | M | RRTRG |
| gene_id "kg2.C_2000021" - | 2 | 287821 | T | C | I | V | NKIGF |
| gene_id "e_gw1.2.1073.1" + | 2 | 296326 | C | A | L | I | RHLIH |
| gene_id "fgenesh5_pg.C_2000094" - | 2 | 318861 | C | G | E | D | DEEYD |
| gene_id "fgenesh5_pg.C_2000094" - | 2 | 318864 | T | G | E | D | DDEEY |
| gene_id "e_gw1.2.729.1" - | 2 | 330088 | T | G | E | A | FTEHN |
| gene_id "e_gw1.2.729.1" - | 2 | 330335 | T | A | T | S | LDTTG |
| gene_id "estExt_GeneWisePlus.C_20163" + | 2 | 336811 | T | C | V | A | ITVRS |
| gene_id "estExt_GeneWisePlus.C_20165" - | 2 | 339757 | G | A | H | Y | TVHQN |
| gene_id "estExt_fgenesh5_pg.C_20103" - | 2 | 348674 | T | A | M | L | SPMQR |
| gene_id "fgenesh1_pm.C_2000039" - | 2 | 359303 | T | C | T | A | LETEA |
| gene_id "estExt_Genewise1.C_20192" - | 2 | 366350 | T | C | I | V | STILL |
| gene_id "estExt_Genewise1.C_20192" - | 2 | 366361 | G | A | P | L | ICPPS |
| gene_id "estExt_GeneWisePlus.C_20184" + | 2 | 368964 | A | G | D | G | EGDFR |
| gene_id "estExt_GeneWisePlus.C_20184" + | 2 | 368970 | G | A | R | K | DFRVL |
| gene_id "estExt_GeneWisePlus.C_20184" + | 2 | 369120 | A | G | D | G | ILDDP |
| gene_id "estExt_GeneWisePlus.C_20184" + | 2 | 369846 | C | A | A | E | RDASR |
| gene_id "estExt_GeneWisePlus.C_20184" + | 2 | 370025 | G | A | G | R | NTGRP |
| gene_id "estExt_GeneWisePlus.C_20184" + | 2 | 370059 | T | C | V | A | PTVDE |
| gene_id "estExt_GeneWisePlus.C_20184" + | 2 | 370074 | A | T | N | I | IRNSI |
| gene_id "estExt_GeneWisePlus.C_20184" + | 2 | 374037 | G | A | G | E | YEGES |

| | | | | | | | |
|---|---|---|---|---|---|---|---|
| gene_id "estExt_GeneWisePlus.C_20184" + | 2 | 374124 | A | C | E | A | VPEGE |
| gene_id "e_gw1.2.750.1" - | 2 | 376789 | C | T | S | N | YLSEW |
| gene_id "e_gw1.2.750.1" - | 2 | 377611 | G | A | P | L | PKPEE |
| gene_id "e_gw1.2.750.1" - | 2 | 377618 | G | A | P | S | EEPKP |
| gene_id "e_gw1.2.750.1" - | 2 | 377663 | T | C | T | A | PVTTG |
| gene_id "e_gw1.2.750.1" - | 2 | 378827 | G | C | L | V | AFLDH |
| gene_id "e_gw1.2.750.1" - | 2 | 379233 | A | C | V | G | SHVNR |
| gene_id "e_gw1.2.750.1" - | 2 | 379744 | T | C | S | G | DNSTF |
| gene_id "e_gw1.2.750.1" - | 2 | 379752 | A | G | L | P | QRLDN |
| gene_id "e_gw1.2.750.1" - | 2 | 379839 | C | T | G | D | SMGSS |
| gene_id "e_gw1.2.750.1" - | 2 | 379841 | C | T | M | I | SSMGS |
| gene_id "e_gw1.2.545.1" + | 2 | 407085 | T | C | F | S | SFFFL |
| gene_id "e_gw1.2.545.1" + | 2 | 407165 | A | G | S | G | KDSNK |
| gene_id "e_gw1.2.545.1" + | 2 | 407286 | G | A | S | N | DESHG |
| gene_id "e_gw1.2.268.1" - | 2 | 426873 | C | A | E | D | DGEKT |
| gene_id "e_gw1.2.740.1" + | 2 | 440129 | T | C | M | T | FPMFG |
| gene_id "estExt_fgenesh5_pg.C_20131" + | 2 | 456359 | A | G | I | V | SFIVQ |
| gene_id "estExt_fgenesh5_pg.C_20131" + | 2 | 457425 | C | T | P | L | SDPRM |
| gene_id "estExt_fgenesh5_pg.C_20131" + | 2 | 457548 | C | T | S | L | GSSRR |
| gene_id "estExt_fgenesh5_pg.C_20131" + | 2 | 457912 | G | C | E | Q | ALELS |
| gene_id "estExt_fgenesh5_pg.C_20131" + | 2 | 458005 | G | A | E | K | SLEFV |
| gene_id "e_gw1.2.796.1" + | 2 | 464376 | G | A | A | T | YYASD |
| gene_id "estExt_fgenesh5_pg.C_20139" + | 2 | 470739 | G | A | V | I | QVVGG |
| gene_id "estExt_fgenesh5_pg.C_20139" + | 2 | 470890 | A | G | H | R | YGHDT |
| gene_id "estExt_fgenesh5_pg.C_20139" + | 2 | 471037 | C | G | A | G | IHALC |
| gene_id "estExt_fgenesh5_pg.C_20139" + | 2 | 471852 | A | G | M | V | AEMVQ |
| gene_id "estExt_fgenesh5_pg.C_20140" - | 2 | 472622 | C | T | D | N | MNDGP |
| gene_id "estExt_fgenesh5_pg.C_20141" + | 2 | 481408 | G | C | G | A | VTGQS |
| gene_id "gw1.2.712.1" - | 2 | 487771 | T | C | N | D | QGNQS |
| gene_id "fgenesh5_pg.C_2000148" + | 2 | 517395 | C | T | P | S | SEPLF |

| | | | | | | | |
|---|---|---|---|---|---|---|---|
| gene_id "fgenesh5_pg.C_2000148" + | 2 | 517575 | A | G | R | G | IPRQD |
| gene_id "fgenesh5_pg.C_2000148" + | 2 | 517605 | C | T | L | F | ASLFR |
| gene_id "fgenesh5_pg.C_2000148" + | 2 | 517875 | G | A | V | I | IRVVP |
| gene_id "fgenesh5_pg.C_2000148" + | 2 | 518133 | A | G | I | V | DVIVD |
| gene_id "fgenesh5_pg.C_2000148" + | 2 | 518164 | T | C | L | S | QSLIL |
| gene_id "fgenesh5_pg.C_2000148" + | 2 | 518194 | C | T | S | F | QDSPA |
| gene_id "fgenesh5_pg.C_2000148" + | 2 | 518426 | C | G | L | V | PFLNI |
| gene_id "fgenesh5_pg.C_2000151" - | 2 | 521124 | A | T | V | E | LRVstopT |
| gene_id "fgenesh5_pg.C_2000151" - | 2 | 521389 | A | C | S | A | ILSAL |
| gene_id "fgenesh5_pg.C_2000151" - | 2 | 521845 | G | A | P | S | DAPRS |
| gene_id "estExt_Genewise1.C_20266" - | 2 | 522419 | C | T | A | T | TTATT |
| gene_id "estExt_Genewise1.C_20266" - | 2 | 522431 | C | T | A | T | SMATT |
| gene_id "estExt_Genewise1.C_20266" - | 2 | 523275 | T | C | K | E | YEKIQ |
| gene_id "estExt_Genewise1.C_20266" - | 2 | 523423 | C | T | M | I | TIMHN |
| gene_id "estExt_Genewise1.C_20266" - | 2 | 523444 | G | T | D | E | VADHT |
| gene_id "estExt_fgenesh5_pg.C_20153" - | 2 | 528460 | C | A | Q | H | FAQFN |
| gene_id "fgenesh1_pm.C_2000057" - | 2 | 529415 | T | C | K | E | MSKDM |
| gene_id "fgenesh1_pm.C_2000057" - | 2 | 531159 | G | T | Q | K | MEQEV |
| gene_id "fgenesh1_pm.C_2000057" - | 2 | 531471 | C | A | V | L | AAVNV |
| gene_id "fgenesh1_pm.C_2000057" - | 2 | 531599 | A | G | L | S | AALQD |
| gene_id "fgenesh1_pm.C_2000057" - | 2 | 532020 | T | C | S | G | PQSGF |
| gene_id "fgenesh1_pm.C_2000057" - | 2 | 532582 | G | T | D | E | PKDGP |
| gene_id "fgenesh1_pm.C_2000057" - | 2 | 532877 | C | T | R | H | QPRQL |
| gene_id "fgenesh1_pm.C_2000057" - | 2 | 533273 | T | G | K | Q | IVKSE |
| gene_id "fgenesh1_pm.C_2000058" + | 2 | 536327 | A | C | K | N | VEKMK |
| gene_id "estExt_fgenesh1_pm.C_20058" - | 2 | 538335 | A | C | D | E | GMDLK |
| gene_id "estExt_fgenesh1_pm.C_20058" - | 2 | 540294 | T | C | I | V | AQIVE |
| gene_id "estExt_GeneWisePlus.C_20271" + | 2 | 558336 | G | C | A | P | PQAQQ |
| gene_id "estExt_GeneWisePlus.C_20271" + | 2 | 558525 | C | A | L | I | DPLAG |
| gene_id "estExt_fgenesh1_pm.C_20062" + | 2 | 564227 | T | C | M | T | MAMCL |

| | | | | | | | |
|---|---|---|---|---|---|---|---|
| gene_id "estExt_fgenesh1_pm.C_20062" + | 2 | 564541 | A | T | K | N | SGKSH |
| gene_id "estExt_fgenesh1_pm.C_20062" + | 2 | 565217 | C | T | P | S | PKPAT |
| gene_id "estExt_fgenesh1_pm.C_20062" + | 2 | 565310 | G | A | G | S | PPGAA |
| gene_id "estExt_fgenesh1_pm.C_20062" + | 2 | 565604 | A | G | T | A | PKTGL |
| gene_id "e_gw1.2.703.1" + | 2 | 572555 | A | C | I | L | SLIEG |
| gene_id "fgenesh5_pg.C_2000167" + | 2 | 577083 | C | T | L | F | FLLSP |
| gene_id "fgenesh5_pg.C_2000167" + | 2 | 577231 | C | A | P | H | NLPFV |
| gene_id "fgenesh5_pg.C_2000167" + | 2 | 577237 | T | C | V | A | PFVNM |
| gene_id "fgenesh5_pg.C_2000167" + | 2 | 578721 | T | G | V | G | SSVTA |
| gene_id "e_gw1.2.689.1" + | 2 | 582435 | C | A | Q | K | RGQQL |
| gene_id "e_gw1.2.689.1" + | 2 | 582630 | A | G | I | V | FEISI |
| gene_id "e_gw1.2.689.1" + | 2 | 583557 | C | T | L | F | GVLDR |
| gene_id "e_gw1.2.689.1" + | 2 | 583704 | G | C | G | R | SRGKD |
| gene_id "e_gw1.2.689.1" + | 2 | 583746 | A | T | I | F | LKIRG |
| gene_id "e_gw1.2.689.1" + | 2 | 585120 | G | A | M | I | VQMKD |
| gene_id "estExt_GeneWisePlus.C_20290" - | 2 | 588778 | G | T | P | T | RIPNI |
| gene_id "estExt_GeneWisePlus.C_20290" - | 2 | 589182 | G | A | S | L | EGSRQ |
| gene_id "fgenesh5_pg.C_2000182" + | 2 | 601459 | T | C | C | R | QVCNQ |
| gene_id "gw1.2.885.1" - | 2 | 605002 | C | T | V | I | TEVAS |
| gene_id "estExt_GeneWisePlus.C_20306" - | 2 | 609790 | G | A | T | I | TLTRN |
| gene_id "estExt_GeneWisePlus.C_20306" - | 2 | 610242 | T | A | L | F | KELLS |
| gene_id "fgenesh5_pg.C_2000185" + | 2 | 612230 | T | G | Y | D | LRYQR |
| gene_id "fgenesh5_pg.C_2000185" + | 2 | 612240 | G | T | R | L | QRRNL |
| gene_id "fgenesh5_pg.C_2000185" + | 2 | 612716 | C | T | P | S | IVPDT |
| gene_id "fgenesh5_pg.C_2000185" + | 2 | 612890 | C | T | P | S | EAPRH |
| gene_id "fgenesh5_pg.C_2000185" + | 2 | 612936 | T | C | F | S | ASFAS |
| gene_id "fgenesh5_pg.C_2000185" + | 2 | 613143 | C | T | A | V | SNAQD |
| gene_id "fgenesh5_pg.C_2000185" + | 2 | 613149 | A | C | D | A | AQDTS |
| gene_id "fgenesh5_pg.C_2000187" - | 2 | 616430 | T | C | D | G | QDDSD |
| gene_id "fgenesh5_pg.C_2000187" - | 2 | 616468 | C | G | E | D | DDEDD |

| | | | | | | | |
|---|---|---|---|---|---|---|---|
| gene_id "estExt_GeneWisePlus.C_20319" - | 2 | 622472 | A | T | F | Y | GGFDA |
| gene_id "estExt_GeneWisePlus.C_20319" - | 2 | 622637 | T | G | D | A | AGDLD |
| gene_id "estExt_GeneWisePlus.C_20319" - | 2 | 622644 | C | A | A | S | LKAGD |
| gene_id "estExt_GeneWisePlus.C_20319" - | 2 | 622920 | C | T | G | S | SPGIL |
| gene_id "estExt_fgenesh1_pm.C_20076" - | 2 | 625399 | A | G | V | A | QEVSL |
| | 2 | 625401 | C | G | E | D | GQEVS |
| gene_id "estExt_fgenesh1_pm.C_20076" - | 2 | 625508 | G | C | L | V | LALEM |
| gene_id "estExt_fgenesh1_pm.C_20076" - | 2 | 625870 | T | C | Q | R | GQQPE |
| gene_id "estExt_fgenesh1_pm.C_20076" - | 2 | 626875 | G | T | P | Q | ASPMS |
| gene_id "estExt_fgenesh5_pg.C_20193" - | 2 | 632019 | T | C | E | G | QLEES |
| gene_id "estExt_fgenesh5_pg.C_20193" - | 2 | 632282 | C | A | L | F | GQLDN |
| gene_id "estExt_fgenesh5_pg.C_20193" - | 2 | 632582 | G | T | A | E | LPAYT |
| gene_id "estExt_fgenesh5_pg.C_20193" - | 2 | 632599 | T | G | E | D | EAEDT |
| gene_id "estExt_fgenesh5_pg.C_20193" - | 2 | 633086 | G | T | T | N | ASTNT |
| gene_id "estExt_fgenesh5_pg.C_20193" - | 2 | 633656 | A | G | W | R | VEWME |
| gene_id "estExt_fgenesh1_pm.C_20077" + | 2 | 634831 | C | G | L | V | AKLGS |
| gene_id "estExt_fgenesh1_pm.C_20077" + | 2 | 635877 | T | A | D | E | QKDEQ |
| gene_id "estExt_fgenesh1_pm.C_20077" + | 2 | 636841 | G | A | A | T | KGAKA |
| gene_id "estExt_fgenesh1_pm.C_20077" + | 2 | 636853 | A | G | T | A | AATKK |
| gene_id "estExt_fgenesh5_pg.C_20195" + | 2 | 637704 | T | A | V | E | EDVAA |
| gene_id "kg2.C_2000050" + | 2 | 640780 | C | G | Q | E | KKQstopG |
| gene_id "fgenesh5_pg.C_2000201" + | 2 | 642880 | A | G | Q | R | HGQST |
| gene_id "fgenesh5_pg.C_2000201" + | 2 | 642926 | T | G | N | K | ITNIH |
| gene_id "fgenesh5_pg.C_2000202" + | 2 | 645421 | A | C | E | D | DQECR |
| gene_id "fgenesh5_pg.C_2000202" + | 2 | 645527 | A | G | R | G | KARAM |
| gene_id "e_gw1.2.303.1" + | 2 | 650685 | G | T | C | F | SMCPK |
| gene_id "e_gw1.2.303.1" + | 2 | 651566 | G | A | V | I | DSVVE |
| gene_id "estExt_GeneWisePlus.C_20334" - | 2 | 655843 | T | G | I | L | PAIAD |
| gene_id "fgenesh5_pg.C_2000206" - | 2 | 659366 | A | G | V | A | EHVAA |
| gene_id "gw1.2.883.1" + | 2 | 661886 | A | G | N | S | ELNVS |

| | | | | | | | |
|---|---|---|---|---|---|---|---|
| gene_id "gw1.2.883.1" + | 2 | 662279 | T | A | I | N | CAIFV |
| gene_id "estExt_GeneWisePlus.C_20346" + | 2 | 667896 | A | G | T | A | AATPA |
| gene_id "gw1.2.1082.1" + | 2 | 672025 | T | C | V | A | SEVNE |
| gene_id "gw1.2.1082.1" + | 2 | 672344 | G | C | Q | H | EKQED |
| gene_id "e_gw1.2.782.1" + | 2 | 674990 | C | A | H | Q | YRHHP |
| gene_id "e_gw1.2.782.1" + | 2 | 675904 | C | T | S | L | ATSSS |
| gene_id "e_gw1.2.782.1" + | 2 | 676812 | A | G | N | D | RLNGA |
| gene_id "e_gw1.2.800.1" - | 2 | 678653 | C | T | V | I | SQVGQ |
| gene_id "e_gw1.2.800.1" - | 2 | 678703 | C | G | S | T | QDSSI |
| gene_id "e_gw1.2.800.1" - | 2 | 678826 | A | G | F | S | NLFAD |
| gene_id "e_gw1.2.800.1" - | 2 | 680123 | A | G | S | P | TESRM |
| gene_id "e_gw1.2.800.1" - | 2 | 680762 | C | G | G | A | YAGAL |
| gene_id "e_gw1.2.173.1" + | 2 | 682185 | C | T | P | S | ETPFA |
| gene_id "e_gw1.2.173.1" + | 2 | 682311 | A | G | T | A | NSTIT |
| gene_id "e_gw1.2.173.1" + | 2 | 682377 | G | A | D | N | IADIR |
| gene_id "e_gw1.2.173.1" + | 2 | 682831 | G | C | G | A | GDGDD |
| gene_id "e_gw1.2.173.1" + | 2 | 682885 | A | G | D | G | QDDDD |
| gene_id "fgenesh5_pg.C_6000390" + | 6 | 1352129 | T | C | F | L | APFIK |
| gene_id "fgenesh5_pg.C_6000390" + | 6 | 1352249 | A | G | T | A | DLTKV |
| gene_id "fgenesh5_pg.C_6000390" + | 6 | 1352778 | A | G | I | V | ANILF |
| gene_id "fgenesh5_pg.C_6000390" + | 6 | 1353295 | G | A | V | I | SNVEA |
| gene_id "fgenesh5_pg.C_6000390" + | 6 | 1353338 | G | A | G | E | AVGLstop |
| gene_id "fgenesh1_pm.C_6000153" + | 6 | 1356859 | C | T | P | S | SNPSA |
| gene_id "gw1.6.51.1" + | 6 | 1360588 | C | T | L | F | PQLQL |
| gene_id "e_gw1.6.418.1" + | 6 | 1367621 | T | A | H | Q | KHHRL |
| gene_id "e_gw1.6.418.1" + | 6 | 1367622 | C | G | R | G | HHRLI |
| gene_id "e_gw1.6.418.1" + | 6 | 1367913 | G | A | V | I | FGVRH |
| gene_id "e_gw1.6.418.1" + | 6 | 1368591 | G | A | V | I | PIVSN |
| gene_id "e_gw1.6.418.1" + | 6 | 1368928 | C | T | S | L | YLSVL |
| gene_id "e_gw1.6.418.1" + | 6 | 1369904 | C | T | P | L | LNPMA |

| | | | | | | | |
|---|---|---|---|---|---|---|---|
| gene_id "fgenesh5_pg.C_6000394" + | 6 | 1373634 | T | C | Y | H | TEYTV |
| gene_id "fgenesh5_pg.C_6000394" + | 6 | 1373873 | G | A | G | E | RMGTE |
| gene_id "fgenesh5_pg.C_6000394" + | 6 | 1373903 | T | C | I | T | KKIDP |
| gene_id "fgenesh5_pg.C_6000394" + | 6 | 1374694 | G | C | R | P | IQRIK |
| gene_id "fgenesh5_pg.C_6000394" + | 6 | 1374729 | G | T | D | Y | NDDAA |
| gene_id "fgenesh5_pg.C_6000394" + | 6 | 1374739 | A | G | Q | R | AAQstopI |
| gene_id "e_gw1.6.269.1" + | 6 | 1384877 | T | A | M | K | DVMPA |
| gene_id "fgenesh5_pg.C_6000397" - | 6 | 1386657 | A | C | S | A | PGSYE |
| gene_id "fgenesh5_pg.C_6000397" - | 6 | 1386894 | G | T | Q | K | KRQSG |
| gene_id "fgenesh5_pg.C_6000397" - | 6 | 1387518 | G | C | Q | E | ARQYL |
| gene_id "fgenesh5_pg.C_6000397" - | 6 | 1387782 | T | C | M | V | LTMRK |
| gene_id "fgenesh5_pg.C_6000397" - | 6 | 1387867 | G | C | D | E | PDDGF |
| gene_id "fgenesh5_pg.C_6000397" - | 6 | 1388364 | G | C | P | A | TPPTT |
| gene_id "e_gw1.6.511.1" - | 6 | 1393434 | A | G | V | A | SLVGD |
| gene_id "e_gw1.6.511.1" - | 6 | 1394368 | G | A | P | S | SAPSL |
| gene_id "e_gw1.6.511.1" - | 6 | 1394666 | A | G | V | A | PAVVV |
| gene_id "e_gw1.6.511.1" - | 6 | 1394734 | C | G | V | L | AKVTG |
| gene_id "e_gw1.6.511.1" - | 6 | 1394885 | A | C | D | E | FHDAA |
| gene_id "fgenesh1_pm.C_6000160" - | 6 | 1403456 | C | G | E | D | AGEED |
| gene_id "fgenesh5_pg.C_6000404" - | 6 | 1407353 | A | C | I | M | PLIHP |
| gene_id "fgenesh5_pg.C_6000404" - | 6 | 1407358 | G | A | L | F | YPLIH |
| gene_id "fgenesh5_pg.C_6000404" - | 6 | 1407422 | C | T | M | I | PPMKQ |
| gene_id "fgenesh5_pg.C_6000404" - | 6 | 1407430 | G | A | P | S | TKPPM |
| gene_id "fgenesh5_pg.C_6000404" - | 6 | 1407517 | G | T | H | N | EPHEH |
| gene_id "fgenesh5_pg.C_6000404" - | 6 | 1407550 | T | G | I | L | SEIIA |
| gene_id "fgenesh5_pg.C_6000404" - | 6 | 1407567 | T | A | N | I | AENAV |
| gene_id "fgenesh5_pg.C_6000404" - | 6 | 1407729 | C | A | R | L | ARRPG |
| gene_id "fgenesh5_pg.C_6000404" - | 6 | 1407765 | C | A | C | F | TTCAI |
| gene_id "fgenesh5_pg.C_6000404" - | 6 | 1408225 | C | T | D | N | IMDYV |
| gene_id "fgenesh5_pg.C_6000404" - | 6 | 1408746 | A | G | M | T | QKMDK |

| | | | | | | | | |
|---|---|---|---|---|---|---|---|---|
| gene_id "fgenesh5_pg.C_6000404" - | 6 | 1409832 | A | G | F | L | DMFSP | |
| gene_id "fgenesh5_pg.C_6000404" - | 6 | 1409852 | T | G | N | T | QLNFD | |
| gene_id "fgenesh5_pg.C_6000404" - | 6 | 1409853 | T | C | N | D | QLNFD | |
| | 6 | 1414830 | T | A | E | V | GIEKV | |
| | 6 | 1414831 | C | T | E | K | GIEKV | |
| gene_id "e_gw1.6.457.1" - | 6 | 1419120 | G | T | P | H | GSPSstop | |
| gene_id "e_gw1.6.457.1" - | 6 | 1419834 | C | A | R | L | SDRLS | |
| gene_id "e_gw1.6.457.1" - | 6 | 1419837 | T | C | D | G | PSDRL | |
| gene_id "fgenesh1_pm.C_6000167" - | 6 | 1434298 | G | A | T | I | AKTNS | |
| gene_id "fgenesh1_pm.C_6000167" - | 6 | 1434425 | T | C | I | V | DEIVQ | |
| gene_id "kg2.C_6000082" + | 6 | 1435527 | T | C | F | L | GAFIY | |
| gene_id "kg2.C_6000082" + | 6 | 1436989 | T | A | F | Y | ELFKA | |
| gene_id "kg2.C_6000082" + | 6 | 1439164 | G | A | S | N | YESKD | |
| gene_id "kg2.C_6000082" + | 6 | 1440050 | C | A | S | R | HASME | |
| gene_id "e_gw1.6.409.1" - | 6 | 1441284 | G | C | S | C | AESKP | |
| gene_id "fgenesh1_pm.C_6000170" + | 6 | 1451674 | G | A | A | T | TKATS | |
| gene_id "fgenesh1_pm.C_6000170" + | 6 | 1451718 | G | C | E | D | GGEFA | |
| gene_id "fgenesh1_pm.C_6000170" + | 6 | 1451893 | G | A | A | T | RPASS | |
| gene_id "e_gw1.21.220.1" + | 21 | 296486 | A | G | N | S | TANTL | |
| gene_id "e_gw1.21.220.1" + | 21 | 297206 | A | R | T | A | GSTLH | |
| gene_id "e_gw1.21.220.1" + | 21 | 297639 | A | M | K | Q | ALKEP | |
| gene_id "estExt_GeneWisePlus.C_210225" + | 21 | 305460 | A | G | K | E | QSKQH | FSF4 |
| gene_id "estExt_GeneWisePlus.C_210225" + | 21 | 305851 | T | C | V | A | KPVEG | |
| gene_id "estExt_GeneWisePlus.C_210225" + | 21 | 305893 | T | C | L | P | GPLAS | |
| gene_id "estExt_GeneWisePlus.C_210225" + | 21 | 305952 | A | C | K | Q | DNKAK | |
| gene_id "estExt_GeneWisePlus.C_210225" + | 21 | 306519 | A | G | M | V | KGMYP | |
| gene_id "estExt_GeneWisePlus.C_210225" + | 21 | 306954 | G | A | A | T | AAATT | |
| gene_id "estExt_GeneWisePlus.C_210225" + | 21 | 307145 | G | C | L | F | KGLKS | |
| gene_id "e_gw1.21.213.1" - | 21 | 310175 | G | A | A | V | PVAGN | FSF5 |
| gene_id "e_gw1.21.213.1" - | 21 | 310397 | T | A | K | M | DGKVP | |

| | | | | | | | | |
|---|---|---|---|---|---|---|---|---|
| gene_id "e_gw1.21.213.1" - | 21 | 311202 | C | T | A | T | PRAAE | |
| gene_id "e_gw1.21.213.1" - | 21 | 311315 | C | T | R | Q | GPRIP | |
| gene_id "e_gw1.21.213.1" - | 21 | 311736 | T | C | T | A | GKTSG | |
| gene_id "e_gw1.21.213.1" - | 21 | 312199 | A | C | D | E | SMDAM | |
| gene_id "e_gw1.21.213.1" - | 21 | 313821 | G | T | H | N | ARHEV | |
| gene_id "estExt_Genewise1.C_210247" + | 21 | 350375 | T | G | D | E | AEDAR | FSF6 |
| gene_id "estExt_Genewise1.C_210247" + | 21 | 351324 | T | C | V | A | ASVAQ | |
| gene_id "estExt_GeneWisePlus.C_210250" + | 21 | 367313 | T | C | S | P | PASTG | FSF8 |
| gene_id "estExt_GeneWisePlus.C_210250" + | 21 | 367316 | A | G | T | A | ASTGD | |
| gene_id "e_gw1.21.222.1" - | 21 | 371812 | T | G | Y | S | RRYLT | FSF9 |
| gene_id "e_gw1.21.222.1" - | 21 | 373476 | C | T | A | T | SSARA | |
| gene_id "e_gw1.21.222.1" - | 21 | 373735 | C | G | M | I | PHMRD | |
| gene_id "e_gw1.21.222.1" - | 21 | 374446 | G | C | L | V | GTLSG | |
| gene_id "estExt_fgenesh5_pg.C_210104" - | 21 | | | | G | S | QRGAL | FSF7 |
| gene_id "e_gw1.21.220.1" + | 21 | | | | | | | |
| gene_id "e_gw1.21.220.1" + | 21 | | | | G | C | VEGEL | |
| gene_id "e_gw1.21.220.1" + | 21 | | | | I | T | VIITF | |
| gene_id "e_gw1.21.220.1" + | 21 | | | | L | F | QSLET | |
| gene_id "e_gw1.21.213.1" - | 21 | | | | D | E | SEDRG | |

Supplementary table 2

**Result of progeny analysis for the presence of selected genes from all three genomic regions which had been retained in all three female fertile strains (Fisher Test).**

The p-values represent probabilities that the presence of the respective gene is correlated with female fertility in the analyzed progeny strain.

| Gene | p-value |
|---|---|
| FS_1 | 1.76E-01 |
| FS_2 | 1.72E-01 |
| FS_3 | 7.98E-03 |
| **FS_4** | **1.30E-06** |
| **FS_5** | **5.03E-09** |
| **FS_6** | **2.00E-07** |
| **FS_7** | **1.08E-08** |
| **FS_8** | **1.59E-09** |
| **FS_9** | **3.47E-09** |
| FS_10 | 1.75E-01 |
| FS_20 | 1.76E-01 |
| FS_21 | 5.91E-02 |
| FS_22 | 3.14E-01 |
| FS_23 | 1.00E+00 |
| FS_24 | 3.50E-01 |
| FS_25 | 8.85E-02 |
| FS_26 | 1.76E-01 |
